# Neutrophils kill the parasite *Trichomonas vaginalis* using trogocytosis

**DOI:** 10.1371/journal.pbio.2003885

**Published:** 2018-02-06

**Authors:** Frances Mercer, Shek Hang Ng, Taylor M. Brown, Grace Boatman, Patricia J. Johnson

**Affiliations:** 1 Department of Microbiology, Immunology & Molecular Genetics, University of California, Los Angeles, Los Angeles, California, United States of America; 2 Molecular Biology Institute, University of California, Los Angeles, Los Angeles, California, United States of America; 3 Pomona College, Claremont, California, United States of America; Princeton University, United States of America

## Abstract

*T*. *vaginalis*, a human-infective parasite, causes the most common nonviral sexually transmitted infection (STI) worldwide and contributes to adverse inflammatory disorders. The immune response to *T*. *vaginalis* is poorly understood. Neutrophils (polymorphonuclear cells [PMNs]) are the major immune cell present at the *T*. *vaginalis*–host interface and are thought to clear *T*. *vaginalis*. However, the mechanism of PMN clearance of *T*. *vaginalis* has not been characterized. We demonstrate that human PMNs rapidly kill *T*. *vaginalis* in a dose-dependent, contact-dependent, and neutrophil extracellular trap (NET)-independent manner. In contrast to phagocytosis, we observed that PMN killing of *T*. *vaginalis* involves taking “bites” of *T*. *vaginalis* prior to parasite death, using trogocytosis to achieve pathogen killing. Both trogocytosis and parasite killing are dependent on the presence of PMN serine proteases and human serum factors. Our analyses provide the first demonstration, to our knowledge, of a mammalian phagocyte using trogocytosis for pathogen clearance and reveal a novel mechanism used by PMNs to kill a large, highly motile target.

## Author summary

The human parasite *Trichomonas vaginalis* is a large unicellular, motile eukaryote that causes a highly prevalent sexually transmitted infection in humans: trichomoniasis. While harmful effects of trichomoniasis are associated with inflammation, the immune response to the parasite is sorely under-characterized. Neutrophils are known to be important players in the host response to *T*. *vaginalis*, but it was not previously known how effective they are at killing the parasite and the mechanism(s) they use to do this. Here, we show that human neutrophils use trogocytosis, a previously undescribed neutrophil mode of microbial killing, to kill *T*. *vaginalis*. Trogocytosis is a process by which a cell takes “bites” of a neighboring cell, a process also referred to as “nibbling.” Using 3D and 4D live imaging, we show that neutrophils rapidly surround and trogocytose *T*. *vaginalis*, prior to parasite death. We rule out whole parasite engulfment (phagocytosis) and the employment of neutrophil extracellular traps (NETosis) in this rapid contact-dependent killing. We also show that antibody–fragment crystallizable (Fc) receptor interactions mediate neutrophil trogocytosis and killing and that serine proteases, commonly employed by neutrophils for microbial degradation, additionally play a role in parasite “nibbling”.

## Introduction

*Trichomonas vaginalis* is a unicellular, flagellated eukaryote that lives as an obligate extracellular parasite, restricted to humans [1,2,3]. *T*. *vaginalis* causes the most common nonviral sexually transmitted infection (STI) worldwide: trichomoniasis [1,4]. The World Health Organization reports approximately 275 million cases each year [1,4]. However, annual reported cases are likely underestimated because the Centers for Disease Control and Prevention estimates that at least 50% of cases are asymptomatic. In the United States, an estimated 1.1 million new infections occur every year [5]. *T*. *vaginalis* is routinely treated with microaerophilic-specific 5-nitroimidazoles: metronidazole and tinidazole [1]; however, metronidazole-resistant strains are on the rise [6,7,8,9,10]. Additionally, *T*. *vaginalis* infection is associated with increased transmission of and susceptibility to HIV, as well as increased progression of cervical cancer in human papilloma virus (HPV)^+^ individuals [11,12,13,14,15,16,17]. *T*. *vaginalis* infection is also associated with bacterial vaginosis, suggesting that infection may disrupt the microflora. Notably, *T*. *vaginalis* was recently classified as a neglected parasitic infection [5] because it disproportionately affects underserved communities [5,18] and contributes to reproductive health disparities; trichomoniasis is linked to pelvic inflammatory disorder, premature and underweight infant birth, infertility, and endometriosis [5,11].

*T*. *vaginalis* complications are thought to have an inflammatory origin. Clinical manifestation of trichomoniasis is associated with an influx of neutrophils—also known as polymorphonuclear cells (PMNs)—to the vaginal mucosa [17]. Studies in preliminary mouse models have shown robust vaginal PMN recruitment after inoculation [19], and human PMNs in vitro were shown to swarm and attack *T*. *vaginalis* [20]. Interleukin 8 (IL-8), a PMN recruitment chemokine [21], is secreted from human vaginal ectocervical cells (Ect-1) [22], human prostate epithelial cells [23], and human monocytes [24,25,26,27] after *T*. *vaginalis* encounter and is found in vaginal secretions of infected patients [25]. PMN involvement during trichomoniasis is likely a major contributor to inflammation-associated pathology because PMNs are potent destructive cells and implicated in other mucosal inflammatory pathologies [21].

PMNs have a diverse arsenal of potent antimicrobial activities and have 3 main modes of killing pathogens: phagocytosis, extracellular degranulation, and casting of Neutrophil Extracellular Traps (NETosis) [21,28]. PMN phagocytosis is aided by pathogen opsonization via antibody and complement [29]. Several types of toxic granules containing numerous effectors, including reactive oxygen species (ROS), pore-forming toxins, elastases, and proteases [30], may be released into the phagosome. When the extracellular degranulation mode of killing is employed, the toxic granules are released extracellularly. Upon activation, PMN granule components work in concert with the NADPH oxidase to achieve H_2_O_2_-mediated oxidative bursts of ROS [31,32]. The use of NETosis results in the release of nuclear contents to form a NET, in which a scaffold of DNA studded with granular components ensnares nearby pathogens. The pathogen is then subsequently engulfed or subjected to toxic granule exocytosis [21,28,33]. The 3 known killing mechanisms used by PMNs are not mutually exclusive, and multiple mechanisms can be used in the attack of a single target.

Recently, PMNs have been shown to perform trogocytosis (trogo = to nibble), a mechanism by which fragments, or “bites,” are taken from one cell by a neighboring cell [34,35,36]. Trogocytosis has been described as a way for immune cells to exchange membrane proteins [34,37,38,39,40,41,42,43] and as a process recently shown to be exploited by intracellular bacteria for cell–cell transfer [44,45]. Parasitic amoebae have also been shown to injure or kill host cells via trogocytosis [46,47,48]. It is notable that immune cell trogocytosis to date has not been demonstrated to be lethal to a pathogen, although there is a recent report linking monocyte trogocytosis to cancer cell death [49].

The mechanisms that PMNs use to control *T*. *vaginalis* have not been examined. Here, we show that PMN killing of *T*. *vaginalis* is mediated in a contact-dependent, NET-independent engulfment process, during which bites of *T*. *vaginalis* are taken by PMNs prior to parasite death, demonstrating a previously undefined role for PMN trogocytosis in pathogen clearance. These studies also reveal a novel mechanism that PMNs can use to kill large, highly motile targets.

## Results

### Human PMNs kill *T*. *vaginalis* in a dose-dependent manner

**Fig 1:**
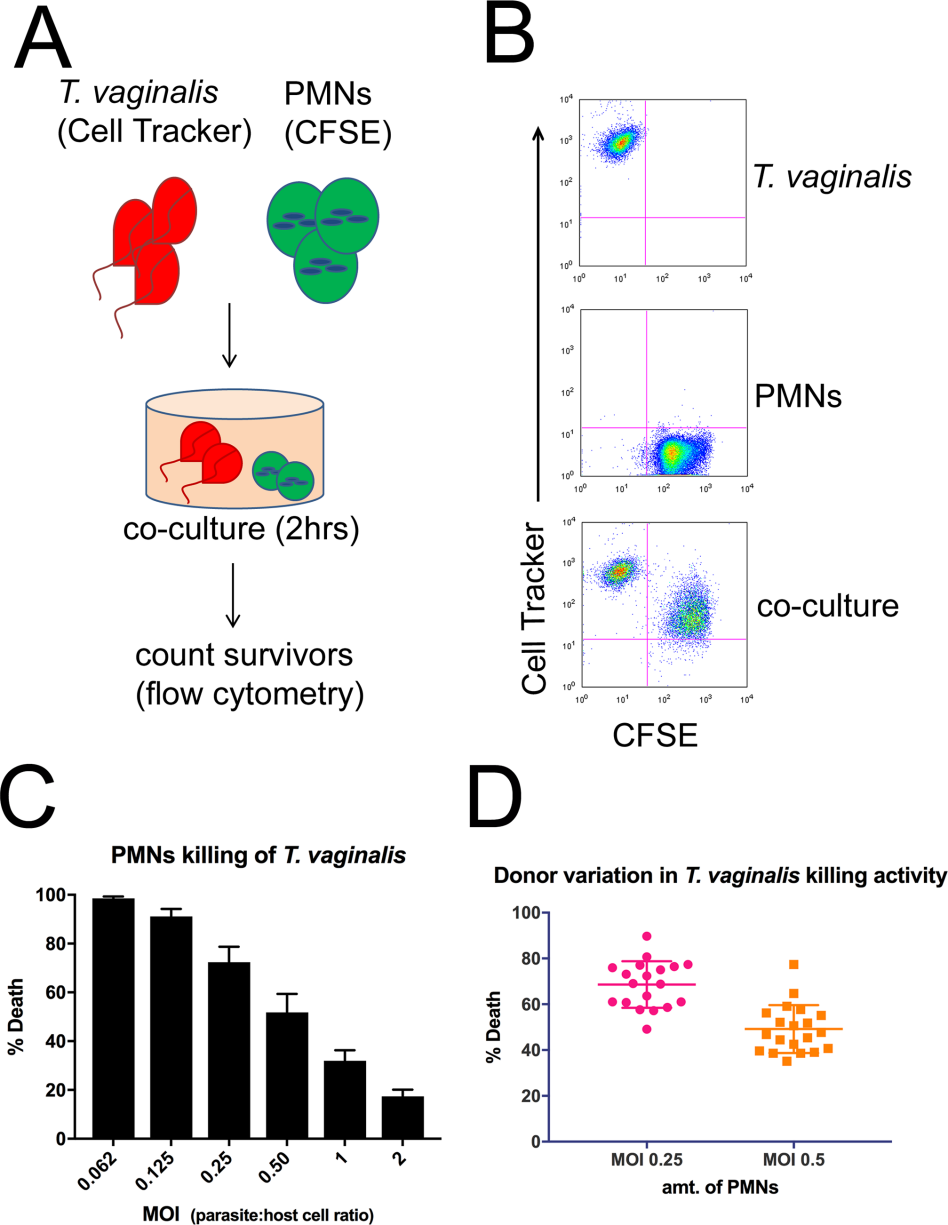
Human PMNs kill *T*. *vaginalis*. (A) Schematic for *T*. *vaginalis* cytotoxicity assay: *T*. *vaginalis* and PMNs were labelled with CT or CFSE, respectively, and cocultured for 2 hours. Cells were then analyzed by flow cytometry to assess the survival of *T*. *vaginalis*. (B) Representative FACS plots of cytotoxicity assay results using an MOI of 0.25 are shown. Surviving *T*. *vaginalis* were defined as CT^+^CFSE^−^. (C) Percent cytotoxicity of *T*. *vaginalis* was determined by counting the number of surviving *T*. *vaginalis* as outlined in A and B, at various MOIs, defined as parasite:host cell ratio. All data are represented as mean ± SD. (D) Composite results of *T*. *vaginalis* cytotoxicity assays, as shown in C, for all 19 donors tested. Each dot represents a different donor. Underlying data can be found in S1 Data and S1, S2, S3, S9 and S10 FCSfiles. CFSE, Carboxyfluorescein succinimidyl ester; CT, Cell Tracker; FACS, fluorescence-activated cell sorting; FCS, fluorescence correlation spectroscopy; MOI, multiplicity of infection; PMN, polymorphonuclear cell.

PMNs have been reported to be abundant during *T*. *vaginalis* infection [1,20], but the mechanism(s) used to kill *T*. *vaginalis* has not been characterized. We therefore employed an in vitro cytoxicity assay to determine whether PMNs can kill *T*. *vaginalis*. In this assay, *T*. *vaginalis* were labeled with Cell Tracker (CT), and PMNs were labeled with Carboxyfluorescein succinimidyl ester (CFSE). Both were then cocultured for 2 hours (Fig 1A). Afterwards, numbers of live *T*. *vaginalis* remaining (CFSE-CT^+^ cells) were quantified to determine whether PMNs are capable of killing *T*. *vaginalis* (Fig 1B, S1 and S2 Figs, and S1–S3 FCS [fluorescence correlation spectroscopy] files). Cell numbers in each condition were normalized to counting beads to ensure that equivalent volumes were analyzed from sample to sample. By comparing the numbers of CFSE-CT^+^ cells in *T*. *vaginalis*–alone conditions to those in which PMNs were present at 1:1 ratio of *T*. *vaginalis*:PMN, we observed an intermediate level of killing (approximately 30%) (Fig 1C and S1 Data). Decreasing the multiplicity of infection (MOI; adding more PMNs) resulted in more efficient killing of *T*. *vaginalis* (Fig 1C), with more than 90% of *T*. *vaginalis* killed at MOI 0.125 (Fig 1C). We found *T*. *vaginalis* killing by PMNs to be reproducible across many different blood donors and the efficiency to be relatively consistent because PMNs from 19 different donors showed similar levels of *T*. *vaginalis* cytotoxicity at MOIs within the dynamic range of our titration (Fig 1D and S1 Data).

### Human PMN killing of *T*. *vaginalis* is contact-dependent and NETosis-independent

**Fig 2:**
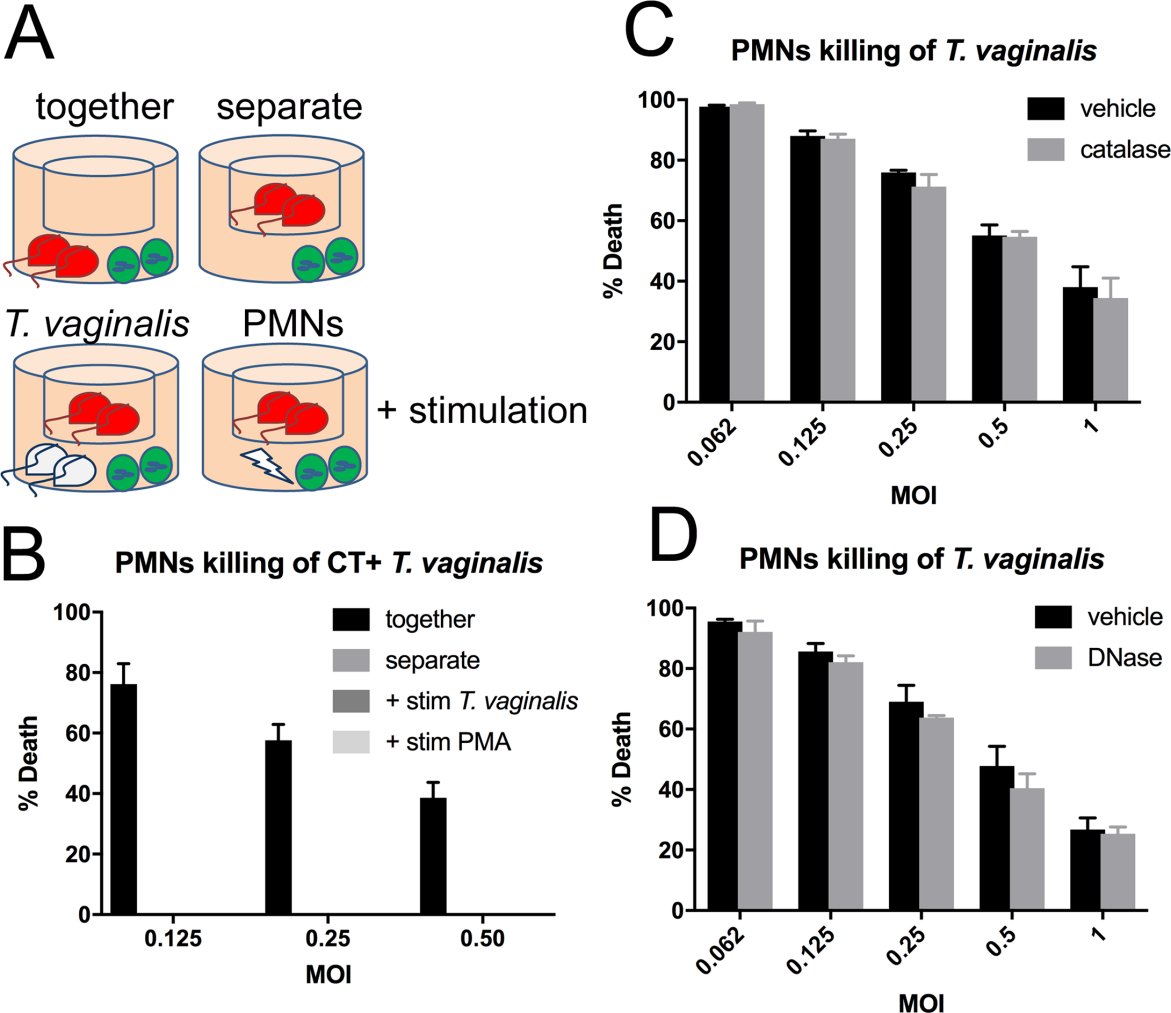
PMN killing of *T*. *vaginalis* is contact-dependent. (A) and (B) *T*. *vaginalis* and PMNs were labelled with CT or CFSE, respectively, and cocultured at the indicated MOI in *trans*-well plates for 2 hours either together in the bottom well or separate, with *T*. *vaginalis* in the top well and PMNs in the bottom well. Cultures were either incubated in the absence or presence of stimulation with 100 nM PMA (lightning bolt symbol) or unlabeled *T*. *vaginalis* (white parasites) added at an equivalent MOI in the bottom well. (C) *T*. *vaginalis* cytotoxicity assays were performed as described in Fig 1, in the presence of (C) 20,000 U/ml catalase or vehicle control (PBS) or (D) 100 U/ml DNase or vehicle control (HBSS). All data are represented as mean ± SD of triplicate wells and representative of 3 donors and 3 independent experiments. CFSE, Carboxyfluorescein succinimidyl ester; CT, Cell Tracker; HBSS, Hank’s Balanced Salt Solution; MOI, multiplicity of infection; PBS, phosphate-buffered saline; PMA, phorbol-myristate acetate; PMN, polymorphonuclear cell.

Because *T*. *vaginalis* is highly motile and slightly larger (10–15 um in diameter) [50] than PMNs (average of 8.85-um diameter in suspension) [51], we next asked whether PMN killing of *T*. *vaginalis* required contact or whether it could be mediated by modes of PMN killing that do not necessarily require contact: extracellular release of toxic granules and ROS or NETosis, a mechanism involving the casting of DNA nets to capture and kill pathogens [52]. To test whether contact-independent degranulation plays a role in PMN killing of *T*. *vaginalis*, we first adapted our *T*. *vaginalis* cytotoxicity assay to a *trans*-well apparatus in which top and bottom chambers share continuous media but are separated by a membrane containing 0.4-um pores (Fig 2A). We found that PMNs were not able to kill *T*. *vaginalis* if they were in separate chambers (Fig 2B and S1 Data). We next asked whether this was due to failure to activate PMNs because *T*. *vaginalis*–PMN contact may be necessary to trigger degranulation. To test this, we compared the killing of labeled *T*. *vaginalis* in the top chamber when PMNs in the bottom chamber were stimulated by either phorbol-myristate acetate (PMA), a standard strong positive stimulus for PMN activation, or were co-incubated with an equivalent amount of unlabeled *T*. *vaginalis* in the bottom chamber (Fig 2A, bottom panel). Both *T*. *vaginalis* and PMA were found to activate PMNs, as assessed by H_2_O_2_ secretion into the media (S3A Fig and S1 Data); however, killing of labeled *T*. *vaginalis* in the top wells was not detected (Fig 2B and S1 Data). To eliminate the possibility that PMN viability was affected in these assays, we assessed viability and found that neither *T*. *vaginalis* nor PMA stimulation affected the viability of PMNs (S3B Fig and S1 Data). To assess the role of H_2_O_2_ in PMN killing of *T*. *vaginalis*, we performed *cis*-well cytolysis assays, coculturing labeled PMNs and *T*. *vaginalis* in the presence of catalase, which inactivates H_2_O_2_ (S4A Fig and S1 Data). We found no abrogation of PMN ability to kill *T*. *vaginalis* (Fig 2C and S1 Data) under conditions in which catalase does not affect PMNs or *T*. *vaginalis* viability (S4B Fig and S1 Data). These data indicate that H_2_O_2_ -mediated oxidative bursts do not play a critical role in *T*. *vaginalis* killing by PMNs.

We next examined whether NETosis contributes to PMN killing of *T*. *vaginalis* by performing our cytolysis assay in the presence of DNase I, which has been shown to degrade NETs and allow pathogen escape [53]. We found that the addition of DNase I, under conditions in which control experiments showed that NETs induced by PMA stimulation of PMNs were degraded (S4C Fig and S1 Data), did not affect the ability of PMNs to kill *T*. *vaginalis* in our *cis*-well assays (Fig 2D and S1 Data). Additionally, DNase I did not affect viability of either cell type in the assay (S4D Fig and S1 Data). These data strongly indicate that NETosis does not contribute to PMN killing of *T*. *vaginalis*.

### PMN killing of *T*. *vaginalis* results in engulfment of *T*. *vaginalis* fragments, as opposed to whole-cell phagocytosis

**Fig 3:**
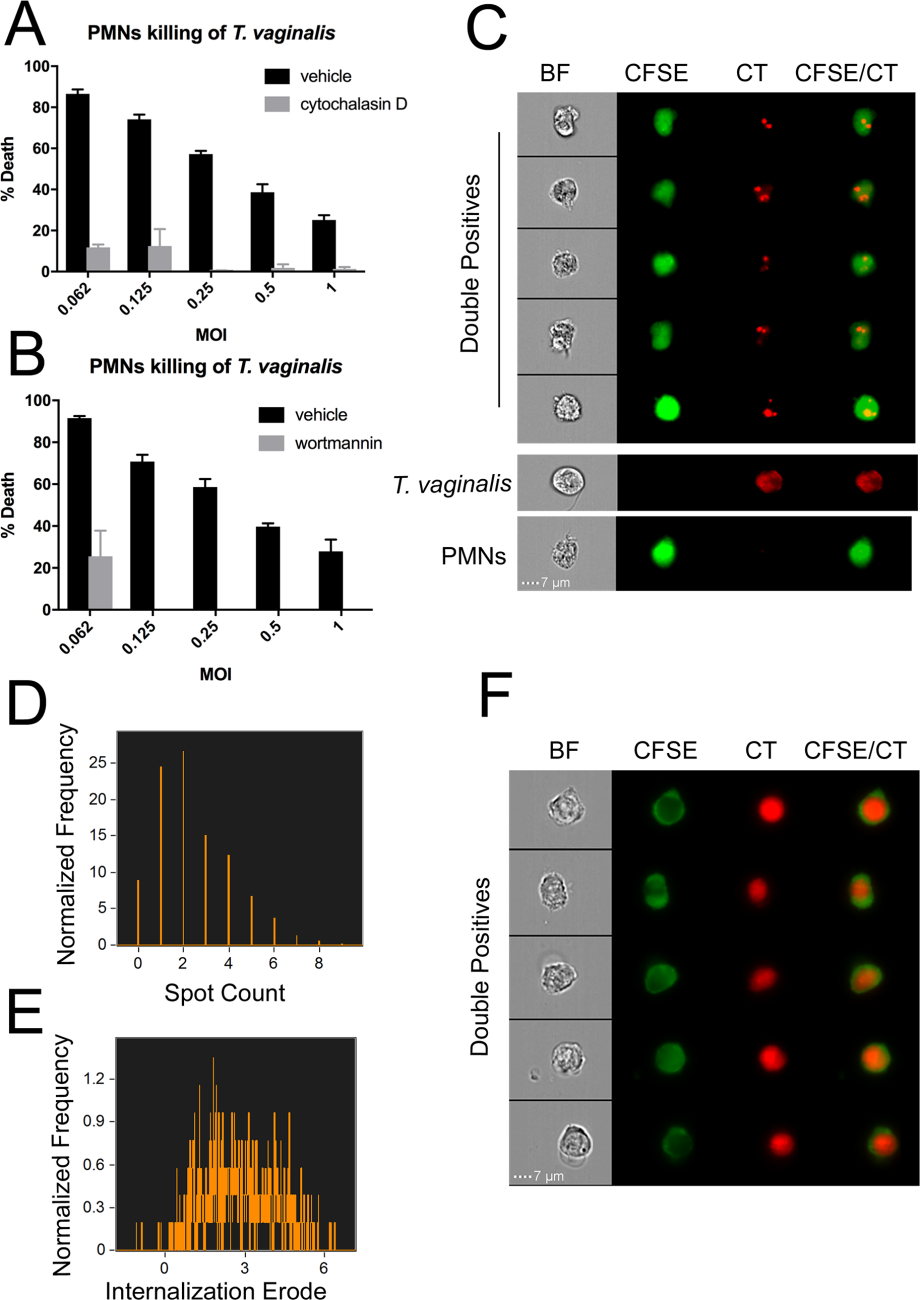
PMN killing of *T*. *vaginalis* involves engulfment. *T*. *vaginalis* cytotoxicity assay was performed as described in Fig 1, except PMNs were pre-incubated with 2.5 ug/ml Cytochalasin D or vehicle control (DMSO) in panel (A) or 50 ng/ml wortmannin or vehicle control (DMSO) in panel (B) for 20 minutes before *T*. *vaginalis* were added. All data are represented as mean ± SD of triplicate wells and representative of 3 donors, and 3 independent experiments. Underlying data can be found in S1 Data. (C) *T*. *vaginalis* and PMNs were labelled with CT or CFSE, respectively, and cocultured for 1 hour. Wells were then harvested, fixed with 4% PFA, and analyzed using Imaging Flow Cytometry at a magnification of 63X. Representative images of CFSE^+^CT^+^ (Double positives), CFSE-CT^+^ (*T*. *vaginalis*), and CFSE^+^CT^−^ (PMN) populations are shown. BF are bright field images. (D) The total double positives population was analyzed for CT^+^ spots within CFSE^+^ cells. (E) Internalization erode score of CT^+^ signal within CFSE^+^ cells was determined for the total double positives population. Internalization erode is the ratio of CT^+^ signal inside of the CFSE^+^ area versus outside. Data are representative of 3 donors and 3 independent experiments. (F) *T*. *vaginalis* were labelled with CT and then incubated at 65 °C for 1 hour and confirmed dead. *T*. *vaginalis* were then cocultured with CFSE-labelled PMNs at identical conditions to that shown in Fig 3, and analyzed by imaging flow cytometry. Representative events from the CFSE^+^CT^+^ double positive gate are shown. BF are bright field images of double positive cells. BF, bright field; CFSE, Carboxyfluorescein succinimidyl ester; CT, Cell Tracker; MOI, multiplicity of infection; PFA, paraformaldehyde; PMN, polymorphonuclear cell.

Taken together, the data shown in Fig 2B demonstrating that PMNs kill *T*. *vaginalis* by a contact-dependent mechanism, and the significant shift of CFSE^+^ cells into the CFSE^+^CT^+^ quadrant suggesting uptake of CT^+^ *T*. *vaginalis* by CFSE^+^ PMNs (Fig 1B), indicate that PMNs may kill *T*. *vaginalis* via engulfment. To test this, we performed our cytotoxicity assay in the presence of inhibitors of actin polymerization and phosphoinositide 3-kinase (PI3K) signaling, both of which have been shown to be necessary for phagocytosis and trogocytosis [36,47,54]. We found that while neither reagent adversely affected the viability of either cell type (S5A Fig and S1 Data), both actin polymerization inhibitor Cytochalasin D and PI3K signaling inhibitor wortmannin significantly decreased PMN killing of *T*. *vaginalis* (Fig 3A and 3B and S1 Data). Treatment with Cytochalasin D and wortmannin also dramatically decreased the occurrence of CFSE^+^CT^+^ double positives (S5B and S5C Fig and S1 Data), consistent with PMN killing *T*. *vaginalis* using engulfment.

Although *T*. *vaginalis* is slightly larger than PMNs, the CFSE^+^CT^+^ double positive population displayed a smear gradient rather than a clear population shift (Fig 1B), inconsistent with PMNs phagocytosing whole parasites. In contrast, these data are consistent with a mechanism called trogocytosis (trogo = to nibble), which PMNs have been recently described to use to capture membranes of neighboring cells [34,35,36]. A related phenomenon, amoebic trogocytosis, has also been shown to be utilized by amoeba to kill host cells [47]. As a first step towards testing whether *T*. *vaginalis* killing proceeds by trogocytosis, a previously undescribed PMN killing mechanism, we visualized CFSE^+^CT^+^ events using Imaging Flow Cytometry. We found that CFSE^+^CT^+^ cells are PMNs that appear to contain fragments of *T*. *vaginalis* (Fig 3C). Using the spot count analysis function of IDEAS software (Seattle, WA), a majority (>90%) of PMNs were found to colocalize with “bites” of *T*. *vaginalis* material at the end of the cytotoxicity assay, and the majority of double positives had between 1 and 4 CT^+^ spots (Fig 3D). Furthermore, the Internalization Erode algorithm of IDEAS software indicated that a vast majority of these spots are internal to PMNs (values >0) (Fig 3E) and not clinging to the circumference of the cell (values <0).

Moreover, to examine whether PMN interaction with *T*. *vaginalis* differs depending on whether the parasite is alive or dead, we compared living and heat-inactivated (dead) *T*. *vaginalis* cocultured with PMNs and found that dead parasites were engulfed intact, i.e., phagocytosed (Fig 3F). We did not observe small spots of *T*. *vaginalis* material in PMNs cocultured with heat-inactivated parasites (Fig 3F and S6A Fig), and cocultures of PMNs with heat-inactivated parasites contained a clear population of CT^+^ CFSE^+^ cells that had equal intensity to parasites alone (CT^+^CFSE^−^) (S6B Fig, S1 Fig, and S4 FCSfile). These data argue against the interpretation that spots of live *T*. *vaginalis* material observed in PMNs result from degradation of parasites that were engulfed whole and instead indicates that bites are taken prior to *T*. *vaginalis* death.

### PMN swarming and trogocytosis of *T*. *vaginalis* precedes *T*. *vaginalis* death

**Fig 4:**
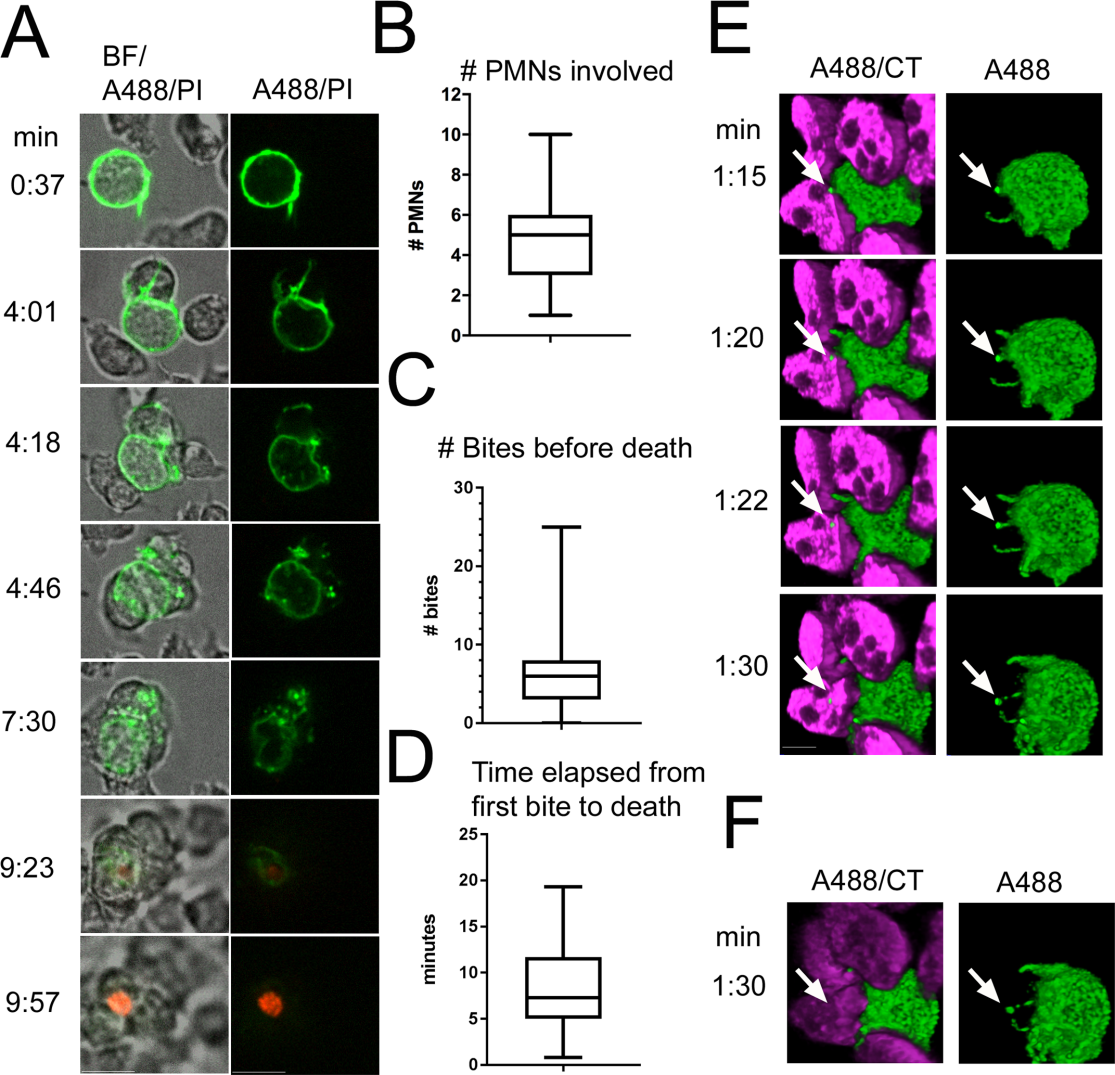
PMNs swarm and trogocytose *T*. *vaginalis* prior to *T*. *vaginalis* death. (A) Live imaging. Total *T*. *vaginalis* surface proteins were covalently linked with Biotin, stained with Streptavidin-Alexa-488 (A488, green), and cocultured with unlabeled PMNs at MOI 0.1 with 10 ug/ml PI in the media. Selected time points of 2D live video at 63X magnification of the interactions are shown. Images are representative of 96 parasite death events performed with PMNs from 11 different human donors. min. = minutes after parasites and PMNs were cocultured. Scale bar = 10 um. (B) and (C) Box and whisker plots showing distribution according to quartiles. (B) 96 videos of *T*. *vaginalis* death events from 11 donors’ PMNs were analyzed for the number of PMNs in contact with *T*. *vaginalis* before the parasite dies. (C) 96 videos from 11 donors’ PMNs were analyzed for the number of “bites” of *T*. *vaginalis* material transferred to PMNs before the parasite dies. (D) Videos in which the first “bite” was observable (19 videos from 7 donors’ PMNs) were analyzed for the time elapsed from when the first bite was taken until PI signal was observed in *T*. *vaginalis*. Underlying data can be found in S1 Data. (E) 3D live imaging. *T*. *vaginalis* surface was labelled with Alexa-488 as in (A) and cocultured with CT-labelled PMNs (magenta) at MOI 0.1 in the presence of 10 ug/ml PI. Z-stacks spanning 15 um were acquired every 1.5 seconds. Selected time points at 63x of PMN engulfment of *T*. *vaginalis* material before parasite death are shown with 3D reconstruction of deconvolved images. Images are clipped midway through the z-axis to visualize the inside of the PMNs. Scale bar = 5 um. White arrow indicates “bite” of *T*. *vaginalis* that is uptaken by PMNs. (F) Last timeframe of (E) is shown without any clipping of the z-axis. Data in E and F are representative of at least 3 replicates each from 3 donors and 3 independent experiments. BF, bright field; CT, Cell Tracker; MOI, multiplicity of infection; PI, Propidium Iodide; PMN, polymorphonuclear cell.

To determine whether “bites” of *T*. *vaginalis* material are found in PMNs before the death of the parasite, we devised a live imaging strategy to monitor cell interactions and cell death. The *T*. *vaginalis* surface was stably labeled with an amine-reactive Biotin and streptavidin Alexa-488 (green), and PMNs were left unlabeled and visualized with bright field imaging. Propidium Iodide (PI; red), a cell-impermeant nucleic acid stain, was added to the media as an indicator of cell death. We found that free-swimming parasites were sought after and surrounded by groups of PMNs (Fig 4A first 3 time points, and S1 & S2 Videos). We then observed transfer and accumulation of green signal into the PMNs (Fig 4A middle 3 time points, and S1 & S2 Videos). At least several minutes later, we observed membrane breach and death of the parasite, as indicated in red (Fig 4A last 2 time points, and S1 & S2 Videos). PI-positive (dead) parasites were clearly discernable from PI-positive (dead) PMNs, the latter having characteristic multilobed nuclei. To rule out the possibility that *T*. *vaginalis* were actively shedding fragments of their surface and PMNs were merely passively endocytosing them, we performed the same experiments with Jurkat cells and never observed any shedding of green material from *T*. *vaginalis* nor appearance of green signal inside Jurkat cells (S7 Fig).

We observed 96 total killings using 11 different human donors’ PMNs and found that generally 3 to 6 PMNs were involved in the swarm before parasite death was achieved (Fig 4B and S1 Data). We found that an average of 3 to 8 “bites” were usually taken from a parasite before its death (Fig 4C and S1 Data), and in some cases, over 20 bites were observed. We also assessed the time that elapsed between the first bite taken and parasite death (becoming PI positive) and determined that in a majority of cases, 5 to 12 minutes passed between the first bite and *T*. *vaginalis* death, with an average of 8.3 minutes elapsed (Fig 4D and S1 Data). These data are inconsistent with “bites” being derived via PMN phagocytosis of dead *T*. *vaginalis* fragments generated through contact-dependent degranulation (CDD). We also observed a small percentage (9%) of parasite deaths occurring before any visible “bites,” which may represent death induced by CDD. Alternatively, it is possible that “bites” were not detected due to limitations of 2D imaging.

Next, to increase our confidence that “bites” of *T*. *vaginalis* material are indeed engulfed by PMNs, we visualized the early stages of PMN–*T*. *vaginalis* interactions using live HyVolution 3D Confocal microscopy (Leica, Wetzlar, Germany). For these experiments, we labeled PMNs with CT, labeled the *T*. *vaginalis* surface with Alexa 488, and added PI to ensure that all parasites were alive at the time of imaging. Only early stages of PMN–*T*. *vaginalis* interactions were visualized because larger PMN aggregates confounded individual PMN boundary discernment. Using 3D reconstruction and Z-clipping to virtually slice through the inside of cells, we found that *T*. *vaginalis* “bites” are internalized by PMNs rapidly after encounter (Fig 4E & 4F and S3 Video).

### Serine proteases mediate PMN trogocytosis and killing of *T*. *vaginalis*

**Fig 5:**
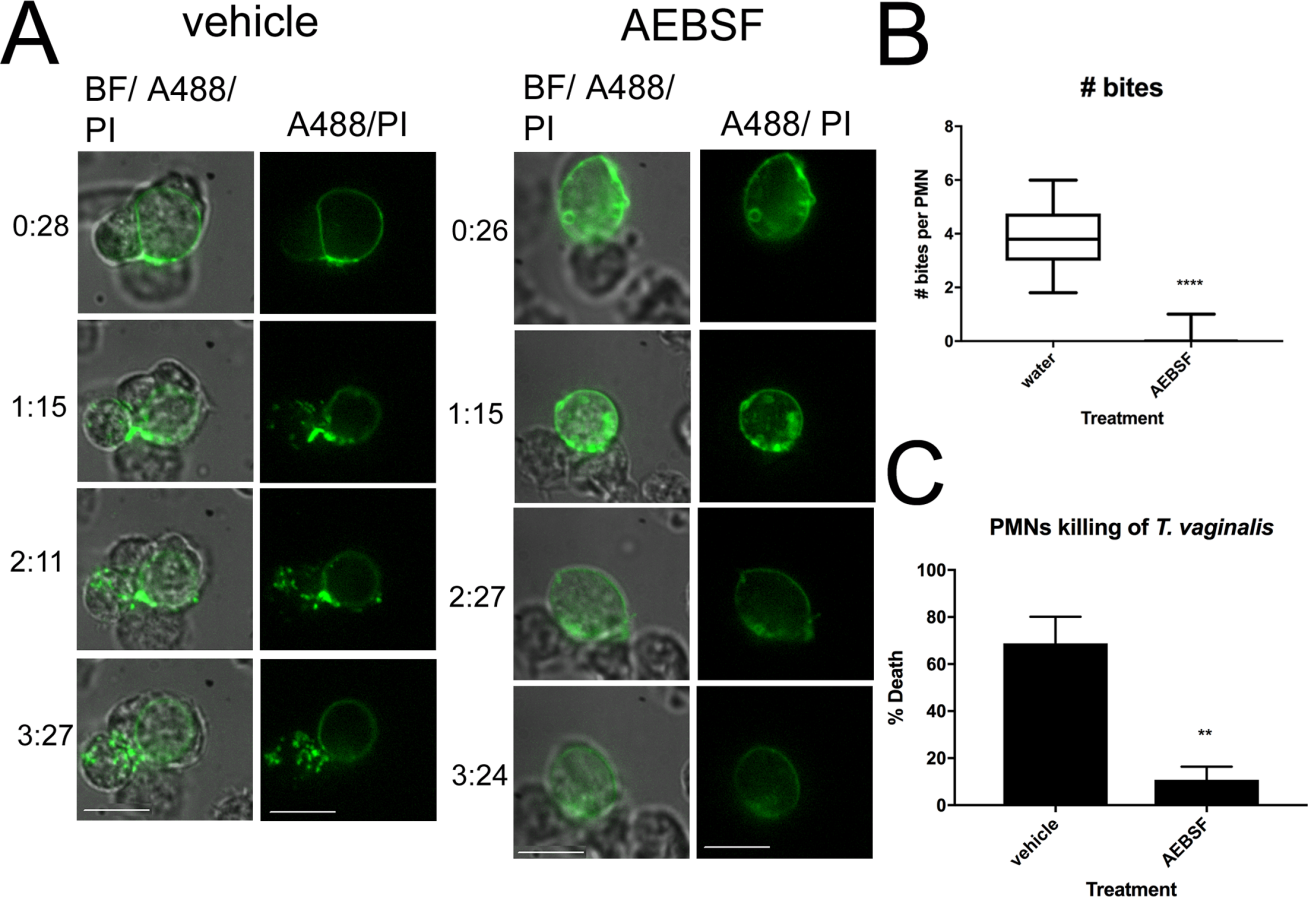
Serine proteases are required for trogocytosis and killing of *T*. *vaginalis*. (A) Live imaging was performed identical to that in Fig 4A, except for the pre-incubation of PMNs either with water (vehicle control) or 1-mM AEBSF for 20 minutes prior to addition of parasites. Scale bar = 10 um. (B) Videos of at least 3 parasites bound to PMNs, chosen at random from 3 donors’ PMNs each were analyzed for the number of “bites” of *T*. *vaginalis* material transferred to PMNs before parasite death, in the presence of vehicle or 1-mM AEBSF, which were pre-incubated with PMNs for 20 minutes before addition of parasites. The number of bites observed was divided by the number of PMNs present in the swarm, in each case. Total number of parasites analyzed was 15 in the vehicle group and 21 in AEBSF. (C) PMN killing of *T*. *vaginalis* at MOI 0.125 (same MOI as panels A and B) was determined using the flow cytometry–based cytolysis assay, in the presence of vehicle or 1-mM AEBSF. Data are represented as mean ± SD of triplicate wells and representative of 3 donors and 3 independent experiments. Underlying data can be found in S1 Data. BF, bright field; MOI, multiplicity of infection; PI, Propidium Iodide; PMN, polymorphonuclear cell.

To assess the mechanism of PMN killing by trogocytosis, we examined the role of PMN proteases in parasite degradation and killing. The most abundant and best-characterized PMN proteases are serine proteases (cathepsin G, neutrophil elastase, proteinase 3, and neutrophil serine protease 4), which mediate major functions in tissue destruction and microbial degradation [55]. Therefore, we tested whether pre-incubation of PMNs with the serine protease inhibitor AEBSF would inhibit PMN trogocytosis of *T*. *vaginalis*. Using live imaging, labeling parasites and PMNs as described above, we observed that AEBSF almost completely inhibited “bites” taken from *T*. *vaginalis* (Fig 5A and 5B and S1 Data). While we did observe reduced swarming motility of PMNs in the presence of AEBSF, consistent with the involvement of serine proteases in chemokine processing [56], PMNs were still observed to bind to and restrain *T*. *vaginalis* (Fig 5A). To prevent artifacts due to reduced swarming, we only imaged parasites that were in direct contact with PMNs and we normalized the number of bites observed to the number of PMNs present in the swarm (Fig 5B). A flow cytometry–based cytolysis assay was also used to assess PMN killing of *T*. *vaginalis* after 2 hours, in the presence of AEBSF. This method confirmed the live imaging data showing that parasite killing was reduced by more than 85% in the presence of AEBSF (Fig 5C and S1 Data). In contrast, phagocytosis of dead (heat-inactivated) *T*. *vaginalis* was only moderately—and not statistically significantly—affected by AEBSF (S8A and S8B Fig and S1 Data), indicating that PMNs are still able to surround and engulf *T*. *vaginalis* in the presence of the inhibitor. Together, these data demonstrate a critical role for PMN serine proteases in the trogocytosis and killing of *T*. *vaginalis*.

### Human serum components facilitate PMN trogocytosis and killing of *T*. *vaginalis*

**Fig 6:**
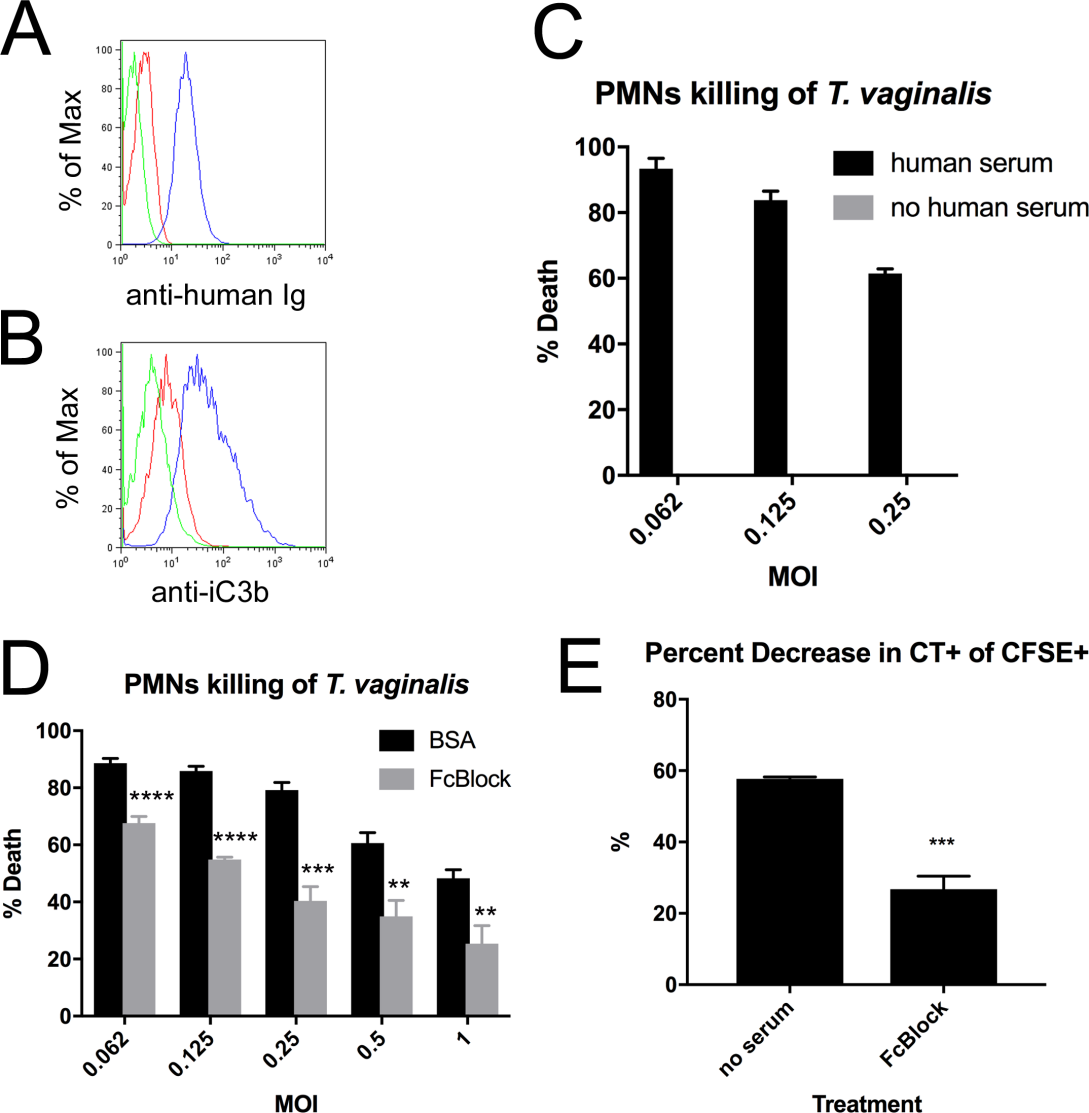
Human serum factors are required for PMN killing of *T*. *vaginalis*. (A, B) *T*. *vaginalis* were stained with 100% human serum, followed by (A) antihuman immunoglobulin light chain kappa or (B) antihuman iC3b, and analyzed with flow cytometry. Green = unstained, red = secondary only (A) or isotype control (B), and blue = fully stained. (C) *T*. *vaginalis* cytotoxicity assay was performed as in Fig 1, either in the presence or absence of 10% human serum. (D) *T*. *vaginalis* cytotoxicity assay was performed in the presence of 8-ug/ml human IgG Fc fragment or equivalent BSA control. (E) Percent decrease in CT^+^ events within total CFSE^+^ events after *T*. *vaginalis* cytotoxicity experiments in (C) and (D). All data are represented as mean ± SD of triplicate wells and representative of 3 donors and 3 independent experiments. Underlying data can be found in S1 Data and S5–S8 FCSfiles. BSA, bovine serum albumin; CFSE, Carboxyfluorescein succinimidyl ester; CT, Cell Tracker; Fc, fragment crystallizable; FCS, fluorescence correlation spectroscopy; MOI, multiplicity of infection; PMN, polymorphonuclear cell.

The killing activity of amoebic trogocytosis requires surface receptor engagement [47]. Trogocytosis by mammalian leukocytes has also been shown to be receptor mediated by antigen receptor-binding interactions in lymphocytes [43] and by antibody–fragment crystallizable (Fc) receptor interactions in phagocytes [36]. To determine whether PMN trogocytosis of *T*. *vaginalis* is mediated by serum factors opsonizing the surface of the parasite in our assay, we first devised staining panels to test whether antibody and iC3b complement factor in our human serum bind to the parasite. We found that both are present on the parasite surface after incubation with human serum (Fig 6A and 6B and S1 Fig, and S5–S10 FCSfiles), consistent with prior reports that serum opsonizes *T*. *vaginalis* [20,57]. We then tested whether removing the 10% human serum in our cytotoxicity assay would affect the ability of PMNs to kill *T*. *vaginalis*. In the absence of human serum, PMNs were unable to kill *T*. *vaginalis* (Fig 6C and S1 Data), and CFSE^+^CT^+^ double positive cells were reduced (Fig 6E and S1 Data), indicating that parasite opsonization is required for optimal trogocytosis and killing. We also found that 10% human serum was required for PMN phagocytosis of dead (heat-inactivated) *T*. *vaginalis*, consistent with a role for human serum factors in *T*. *vaginalis* opsonization (S8C Fig and S1 Data). Finally, we performed our *T*. *vaginalis* cytotoxicity assay in the presence of Fc receptor–blocking agent and found that *T*. *vaginalis* killing was decreased moderately overall and by around 50% in the dynamic range of the assay (MOI 0.25) (Fig 6D and S1 Data), consistent with antibody opsonization mediating PMN cytotoxicity towards *T*. *vaginalis*. We similarly observed a decrease in CT^+^CFSE^+^ cells in cocultures treated with Fc receptor–blocking agent (Fig 6E and S1 Data), demonstrating a role for Fc receptors in trogocytosis of *T*. *vaginalis*. These effects were not due to adverse effects of the Fc-blocking agent on *T*. *vaginalis* or PMNs alone because neither affected viability (S9 Fig and S1 Data).

## Discussion

Despite the high prevalence and adverse inflammatory outcomes of *T*. *vaginalis* infection, the immune response to this parasite has been poorly characterized. PMNs are known to be major mediators of inflammation and are abundant at the sight of *T*. *vaginalis* infection [1,17], but neither their effectiveness nor the mechanism(s) used to kill *T*. *vaginalis* have been previously examined. Here we show that the killing of *T*. *vaginalis* by human PMNs involves cooperative swarming of PMNs around the parasite, followed by attachment and trogocytosis, which precedes parasite death. This novel mechanism for killing a pathogen is mediated by antibody–Fc receptor engagement and PMN serine protease degradation of *T*. *vaginalis* into “bites”.

Trogocytosis is a process by which cells take “bites” of neighboring cells. It has been described to be used by immune cells [37,38,39,40,41,42,43], embryonic cells during development in *Caenorhabditis elegans* [58,59], and by amoebae [46,47,48]. Trogocytosis by *Entamoeba histolytica* has been shown to result in death of mammalian target cells [47,60,61,62], and trogocytosis between immune cells has been shown to be exploited as a conduit for transfer of the intracellular bacterium *Francisella tularensis* from cell to cell while evading the immune system [44,45]. More recently, PMNs have been shown to trogocytose tumor cells coated with monoclonal antibody (mAb) [35], and macrophages have been shown to kill tumor cells using trogocytosis [49]. The data presented here represent, to our knowledge, the first demonstration of mammalian phagocyte trogocytosis being used to kill a pathogen.

Humans are the only hosts infected by *T*. *vaginalis*. To date, attempts to establish robust mouse models with reproducible ability to infect and that sustain adequate *T*. *vaginalis* titers to allow reliable analysis of immune function have failed. The most promising model required a combination of estrogen and dexamethasone treatment to allow infection [19], both of which are functionally suppressive to PMNs, and generally immunosuppressive [63,64,65], placing severe limitations on the use of mouse models to study the immune response to *T*. *vaginalis*. In lieu of an animal model, we have used PMNs derived from human donors to characterize the interaction and killing of *T*. *vaginalis* by PMNs. We found that PMN killing of *T*. *vaginalis* is most efficient at higher PMN:*T*. *vaginalis* ratios (Fig 1C), consistent with our observations using live imaging that PMNs swarm around the parasite (Fig 4A), and that more than one PMN was needed to achieve killing, with rare exceptions (Fig 4B). In vaginal discharge from trichomoniasis patients, the PMN:*T*. *vaginalis* ratio was measured at greater than 100:1 [20], indicating that PMNs likely outnumber *T*. *vaginalis* in vivo, increasing confidence that these in vitro findings are physiologically relevant. Furthermore, prior studies with lower-resolution imaging techniques and light microscopy of vaginal smears from infected patients have observed “membrane fusion” between *T*. *vaginalis* and PMNs from infected patients [66]. Likewise, ex vivo studies of parasites cultured with human PMNs have described “killing by fragmentation” [20]. Both of these observations are consistent with killing using trogocytosis.

We found no evidence of PMN killing of *T*. *vaginalis* by NETosis by comparing parasite death in the absence and presence of DNase (Fig 2C). These data were corroborated by live imaging data that reveal that killing of *T*. *vaginalis* by PMNs was typically completed within approximately 15 minutes, whereas NETosis is generally a later-stage PMN killing mechanism, optimally assessed after 4 hours [67]. Furthermore, although our live imaging experiments contained an extracellular nucleic acid stain, no NETs were detected during imaging.

PMN killing of *T*. *vaginalis* was found to be contact dependent (Fig 2B), indicating that extracellular degranulation of toxic granules is not sufficient for *T*. *vaginalis* killing. It is possible that *T*. *vaginalis* can inactivate granular components or that the parasite is robust enough to withstand assaults from the granules because *T*. *vaginalis* secretes myriad proteases thought to play a role in virulence [3,68,69,70,71,72,73]. However, soluble granule components may not be toxic to *T*. *vaginalis* at dilute concentrations but may need to be secreted directly onto the parasite in a synapse, similar to antibody-dependent cell-mediated cytotoxicity (ADCC) or other receptor-mediated CDD [29]. In this regard, it is notable that PMN serine proteases were found to be critical for both trogocytosis and killing of *T*. *vaginalis* (Fig 5). Although PMN serine proteases are found at highest concentrations in the granules, they also localize to the plasma membrane upon PMN activation and can act in sealed compartments [55]. Therefore, a possible mechanism for PMN-mediated trogocytosis and death of *T*. *vaginalis* may involve recruitment of granules to the parasite surface, allowing proteases within the granules to nibble, taking “bites” of the parasite. Alternatively, serine proteases may be relocalized to the plasma membrane, where they are able to nibble within the tight microenvironment of the PMN–*T*. *vaginalis* junction. Such a killing mechanism would employ CDD in strict conjunction with trogocytosis. Future studies should elucidate whether this is a general mechanism used for PMN killing of larger pathogens and targets that otherwise escape phagocytosis and are resistant to contact-independent extracellular degranulation.

Our observation that bites of *T*. *vaginalis* material usually occur and accumulate in PMNs at least several minutes before parasite death (average of 8.3 minutes) indicates that trogocytosis proceeds PMN killing of *T*. *vaginalis* (Fig 4D). The accumulation of “bites” inside PMNs before parasite death argues against the interpretation that parasites are being killed by serine proteases and then PMNs are merely “cleaning up” the fragments through phagocytosis. The number of bites observed prior to *T*. *vaginalis* death was variable, averaging 3 to 8, but as many as 25 bites were observed (Fig 4C). Induction of a wound-repair mechanism during early stages of trogocytosis may allow parasite survival but may become overwhelmed eventually. The observation that death of some parasites occurs after only a few bites whereas others can survive up to 25 bites indicates PMN killing of *T*. *vaginalis* is multifactorial—consistent with a role for CDD, dependent on the number of PMNs in the swarm surrounding the parasite and other variables. Number of bites required for death may also be dictated by whether bites are initially taken from the flagellum or the plasma membrane in closer proximity to the cytosol.

Several parallels exist between the lethal trogocytosis mechanism of PMN killing of *T*. *vaginalis* described here and amoebic trogocytosis described for the killing of host cells by the parasite *E*. *histolytica* [47]. Both mechanisms result in punctate “bites” of the target cell appearing in the effector cell, which precede the death of the target cell. Both also require live bait for trogocytosis because feeding dead cells to effectors results in whole-cell engulfment of the target (Fig 3F & S6 Fig) and both are receptor mediated (Fig 6) [47,48]. Interestingly, the surface molecules that mediate contact during trogocytosis also mediate contact between effector and target cells for other purposes. In the case of *T*. *vaginalis*, human serum factors are also necessary for phagocytosis of dead parasites (S8 Fig). We therefore conclude that receptor-mediated surface contact is necessary, but not sufficient, for trogocytosis. The similarity in killing mechanisms used by these very divergent eukaryotic cell types is remarkable and suggests that the ability of cells to trogocytose neighboring cells evolved early in eukaryotic evolution and may be more widespread and common than currently acknowledged.

Anti–*T*. *vaginalis* antibodies are present in sera of infected patients [74,75], and cysteine proteases secreted by *T*. *vaginalis* have been shown to degrade human immunoglobulin [71]. We have also shown *T*. *vaginalis* to be preferentially cytotoxic to B cells using contact-dependent and contact-independent mechanisms [27]. *T*. *vaginalis* may therefore have evolved strategies to evade humoral immunity in order to avoid rapid clearance by PMNs. This is consistent with partner reinfection of trichomoniasis being common [76], indicating ineffective adaptive immunity. It will be important to determine whether trogocytosis and PMN killing of *T*. *vaginalis* strictly requires the presence of cognate antibody or whether innate factors in serum such as complement can also mediate killing and trogocytosis. iC3b was observed to bind to the surface of *T*. *vaginalis*, and Fc-blocking treatment only partially inhibited PMN cytolysis of *T*. *vaginalis* (Fig 6), indicating that antibody-independent mechanisms may also mediate trogocytosis and killing.

PMNs are also important in mediating therapeutic effects of directed mAb therapy against cancers. PMN surface Fc receptors can bind opsonized tumor cells, which can then be eliminated by ADCC or phagocytosis [77]. However, recent reports have also attributed failings of some mAb treatments to PMN trogocytosis [78]. Using trogocytosis, phagocytes containing Fc receptors have been shown to clip off exogenous mAbs from the surface of tumor cells, effectively enabling therapeutic escape and cancer cell survival [36,78]. The reason or rationale for this is unclear. A host defense role for PMN trogocytosis in pathogen clearance, demonstrated here, could help explain Fc receptor–mediated trogocytosis by phagocytes.

These data indicate that human PMNs swarm, trogocytose, and kill *T*. *vaginalis*, a process that is enhanced by adaptive immunity (antibody) and is dependent on PMN serine proteases. Further characterization of the specific molecular determinants of this process should better inform vaccine strategies and immunotherapies to mitigate adverse inflammatory complications resulting from *T*. *vaginalis* infection. Future studies are also likely to illuminate whether this novel mechanism of lethal PMN trogocytosis is broadly used to combat large, motile pathogens.

## Materials and methods

### Ethics statement

All human blood material was obtained from the University of California, Los Angeles (UCLA) Center for AIDS Research (CFAR) Virology Core Facility. Use of this material was approved by UCLA Institutional Review Board (IRB) committee Medical IRM 2, protocol 11–000443. All participants were 18 years of age or above and gave written consent.

### Isolation of human PMNs

Blood was obtained periodically at random from among approximately 50 de-identified healthy donors from the UCLA Virology Core. Peripheral Blood Mononuclear cells were removed after Ficoll gradient, and the remaining pellets were subject to a 3% Dextran (Pharmacosmos, Denmark) in 0.9% NaCl gradient for 20 minutes. The top layer was isolated and spun at 250 g for 10 minutes. The pellet was then treated with 20 mL ACK lysing buffer (Invitrogen; Thermo Fisher Scientific, Carlsbad, CA) to remove residual erythrocytes, and resulting PMNs were washed in PBS and stored on ice. PMNs were confirmed to be more than 98% viable and pure as assessed by flow cytometry scatter and dead cell exclusion using Zombie Red viability dye (Biolegend, San Diego, CA), CD11b antibody staining, and microscopy for PMN nuclear morphology using DAPI. Experiments with PMNs were always commenced immediately or within 1 hour of isolation.

### *T*. *vaginalis* strains and culture

*T*. *vaginalis* strain G3 (Beckenham, UK 1973, ATCC-PRA-98) was grown as described [27]. Briefly, parasites were maintained in supplemented TYM medium [79] at 37 °C and maintained at approximately 1 x 10^5^ to 2 x 10^6^ cells/mL. To ensure that all strains used in this study were free of the common symbiont *Mycoplasma hominis* [80], media was supplemented with 50 μg/ml chloramphenicol and 5 μg/ml tetracycline (Sigma-Aldrich, St. Louis, MO), supplemented for at least 5 days, as described previously [27]. Dead, intact controls were generated as described [27]. Briefly, *T*. *vaginalis* were reconstituted in complete RPMI media and heated at 65 °C for 1 hour. Parasites were confirmed by live microscopy to be immobile but intact as described [27], and loss of viability was confirmed with PI (Biolegend).

### PMN–*T*. *vaginalis* coculture conditions

Coculture experiments were performed as described [27]. Briefly, PMNs and *T*. *vaginalis* were combined in RPMI 1640 media and incubated at 37 °C with 5% CO_2_. Ten percent human AB serum from the same batch (Lot 35060105, Corning, Corning NY) was thawed on ice and supplemented into media at the time of coculture for all experiments, except the human serum-deficient condition in Fig 6.

### *T*. *vaginalis* cytotoxicity assay

Flow cytometry–based cytotoxicity assays were utilized, as described previously [27]. Briefly, *T*. *vaginalis* were labeled with either CT Blue or Red (Molecular Probes) and recovered in complete TYM media for 45 minutes to 2 hours. *T*. *vaginalis* was then resuspended in RPMI, and 1.5 x 10^4^ parasites were added to each well of a 96-well u-bottom plate. CFSE-labeled PMNs were added to the plate at the indicated MOI and analyzed by flow cytometry. Counting beads (Life Technologies, Carlsbad, CA) were added to each sample before analysis. Counts of parasites and CFSE^+^CT^+^ double positives were determined as in Fig 1B. Percent *T*. *vaginalis* killing was calculated as ([number of *T*. *vaginalis* in *T*. *vaginalis*–alone condition − number of *T*. *vaginalis* in coculture condition] / number of *T*. *vaginalis* in *T*. *vaginalis*–alone condition) x 100. Zombie Red dead-cell exclusion dye (Biolegend) was added to preliminary experiments to ensure that live cell gates did not include dead cells, and then found to be redundant because dead cells completely disappear from the live cell scatter plots (S2 Fig) and was no longer included, for simplicity. *Trans*-well cytotoxicity experiments were also conducted as described [27]. Briefly, a *trans*-well plate with 0.4-μm polycarbonate membrane was utilized, and PMNs were placed in the bottom chamber. After the cocultures, both top and bottom chambers were harvested. Where noted, inhibitors/activators were added to *T*. *vaginalis* cytotoxicity assays either at the time of coculture (DNase-1 [Worthington], Catalase [Sigma], PMA [Cayman Chemical]), to PMNs 10 minutes before addition of *T*. *vaginalis* (Human IgG F(c) fragment [Rockland Chemical]), or to PMNs 20 minutes before the addition of *T*. *vaginalis* (Cytochalasin D [Sigma], wortmannin [Tocris], AEBSF [Tocris]).

### Determination of PMN and *T*. *vaginalis* viability

For quality control experiments, *T*. *vaginalis* viability was determined by labelling with CT and counting intact cells on flow cytometry as described above. Because PMNs adhere to plates after activation, the use of counting by flow cytometry was ineffective for determining viability, and instead viability was assessed using cytosolic mammalian-specific lactate dehydrogenase (LDH) detection in supernatants using the CytoTox-One homogeneous membrane integrity assay (Promega, Madison, WI) as described [27]. Briefly, 5 x 10^5^ PMNs were plated in 100 ul in a 96-well u-bottom plate, and after treatment, supernatants were assayed for LDH. Percent death was calculated as described [27]. Inhibitors were added at the same concentration and timeframe as in *T*. *vaginalis* cytotoxicity assays.

### H_2_O_2_ detection

The Amplex Red extracellular H_2_O_2_ detection kit (Invitrogen) was used according to the manufacturer’s instructions with 2.5 x 10^5^ PMN/well in a 96-well plate, with final volume 100 ul. After Amplex Red and Horseradish peroxidase were added, the plate was incubated for 2 hours at 37 °C, 5% CO_2_ and then read on a Victor^3^ 1420 plate reader (Perkin-Elmer, Waltham, MA). To determine Catalase inhibition, Catalase was added at the beginning of the assay.

### Extracellular DNA (NET) detection

NET release from PMNs was detected using the picogreen extracellular DNA quantitation kit (Life Technologies) according to the manufacturer’s instructions. A total of 2.5 x 10^5^ PMNs were plated in 96-well flat-bottom plates and stimulated with 100-nM PMA to serve as a positive control for NET induction. After 2 hours, 50 ul of supernatant was harvested and read on a Victor^3^ 1420 plate reader (Perkin-Elmer), after 2-minute incubation with picogreen.

### Image stream analyses

Samples were run on either an Amnis FlowSight or ImageStream Mark II. A total of 5,000 events were collected for each sample. Single color controls were used for auto compensation by IDEAS software. Analysis was done only on cells in focus, and doublets and debris were eliminated based on area versus aspect ratio. Spot Count, Internalization Erode, Intensity, and circularity algorithms were done in using standard wizards in IDEAS software. Internalization positive scores were generated using the Internalization Erode algorithm with CT as internalizing probe and CFSE as cell boundary. For phagocytosis analyses, percent of *T*. *vaginalis* phagocytosed was calculated as follows: ([number of CT^+^CFSE^+^ cells] / [Total number of CT^+^ cells]) x (percentage of CT^+^CFSE^+^ cells that are internalization positive / 100).

### Preparation of cells for live imaging

Parasites were washed 2 times in 5% sucrose PBS and incubated for 45 minutes with cell-impermeant Sulfo-NHS-SS-Biotin (Thermo Fisher Scientific) in 5% PBS sucrose to label amine groups on surface proteins. The reaction was quenched for 5 minutes on ice in 50 uM Tris pH 7.5, and then parasites were washed 3 times with 5% PBS sucrose and incubated on ice for 5 min with 5 ug/ml Streptavidin-Alexa 488 (Biolegend). Parasites were then washed 2x with 5% sucrose PBS, counted, resuspended in RPMI, and stored on ice. For experiments in Fig 4E and 4F, PMNs were labeled with CT Deep Red and stored on ice.

### 2D live imaging

Two-dimensional live imaging videos were taken using a Zeiss confocal microscope outfitted with Yokogawa spinning disc confocal scanner unit and Oko lab 37-degree chamber, as assembled by Intelligent Imaging Innovations (3i, London, UK). SlideBook6 software was used for acquisition and analysis. A total of 1.3 x 10^6^ PMNs were plated in RPMI with 10% human serum in a 35-mm MatTek dish and allowed to settle down to the glass bottom for 15 minutes. Then, 1.3 x 10^5^ *T*. *vaginalis* were added slowly in a dropwise fashion around the plate, and video was commenced once parasites reached the PMN layer. Sequential exposures with BF, 488, 561, and 640 nm were taken approximately every 0.5 seconds, and x, y, and z parameters were adjusted by hand during the video to follow individual parasites and trogocytic events. Videos shown are at 0.1 frame/second. For composite data (Fig 4B and 4C), some videos were taken at 40x to visualize more parasites/field, and some were taken at 63x.

### 3D live imaging

Three-dimensional live imaging videos were taken using the same plating strategy as for 2D imaging, but HyVolution super resolution acquisition was done on a Leica Sp8 Scanning Confocal microscope in a 37-degree chamber. Z-sections were taken every 0.3 um over an approximately 20-um section, set by hand depending on parasite and PMN position. Using the white laser and HyD sensors, 488 and 632 wavelengths were excited and collected simultaneously, and 561 was done sequentially, approximately every 2 seconds/time point. Data were then deconvolved using Huygen’s Professional (Scientific Volume Imaging, Hilversum, Netherlands), and processing and 3D reconstruction were done in LASX software (Leica, Wetzlar, Germany). Video shown is 0.25 frames/second.

### *T*. *vaginalis* staining with human serum

Human serum Lot 35060105 (Corning, Mediatech) was thawed on ice, and *T*. *vaginalis* were immediately incubated with serum for 30 minutes at 37 degrees. *T*. *vaginalis* were then washed 2x with FACS buffer (1x PBS, 5% FBS, 0.1 NaN_3_), and the following staining steps were done for 30-minute incubations on ice with FACS buffer washes in between. For detection of human antibody bound to *T*. *vaginalis*, parasites were incubated on ice for 30 minutes with either PE antihuman immunoglobulin light chain kappa or isotype control (Biolegend). For detection of iC3b on *T*. *vaginalis*, parasites were incubated with purified mouse antihuman iC3b or isotype control, followed by PeCy7 antimouse IgG1 (Biolegend). Parasites were then stored on ice and analyzed on LSR Fortessa within 2 hours.

### Statistics

All statistical analyses were done using a two-tailed, unpaired *t* test in Microsoft Excel (Redmond, WA).

## Supporting information

S1 Fig Gating strategies for all FACS plots. Gating strategies defining populations shown in FACS plots in Figs 1B, 6A and 6B and S6B using FCS files available as S1–S10 FCSfiles are shown. FACS, fluorescence-activated cell sorting; FCS, fluorescence correlation spectroscopy.
(TIF)

S2 Fig CT^+^CFSE^−^ cells remaining after 2-hour coculture with PMNs are viable. (A) To ensure that our live cell gate was accurate for flow cytometry–based cytolysis experiments, we labelled cells and cocultured as described in Fig 1. Then, all wells were stained with Zombie Red dead cell exclusion dye. The Zombie Red^+^ gate was set based on live *T*. *vaginalis* alone. We observed ≥98% of cells to be Zombie Red negative at all MOIs tested, indicating that surviving parasites in our assays are viable. CFSE, Carboxyfluorescein succinimidyl ester; CT, Cell Tracker; MOI, multiplicity of infection; PMN, polymorphonuclear cell.
(TIF)

S3 Fig Quality controls for *T*. *vaginalis* cytotoxicity assays performed in a *trans*-well assay. (A) PMN degranulation, as assessed by Amplex Red indicator of H_2_O_2_ activity, in the presence of *T*. *vaginalis* MOI 0.125 or 100 nM PMA, is shown. (B) PMNs (black) and *T*. *vaginalis* (grey) were incubated for 2 hours in the presence of *T*. *vaginalis* MOI 0.125 or 100 nM PMA, and viability was determined as described in Materials and methods. All data are represented as mean ± SD of triplicate wells and representative of 3 donors and 3 independent experiments. Underlying data can be found in S1 Data. MOI, multiplicity of infection; PMA, phorbol-myristate acetate; PMN, polymorphonuclear cell.
(TIF)

S4 Fig Quality controls for *T*. *vaginalis* cytotoxicity assays done with DNase and Catalase. (A) H_2_O_2_ secretion, as assessed by Amplex Red indicator, was measured in wells of PMNs treated with MOI 0.125 *T*. *vaginalis* with or without 20,000 U/ml Catalase. (B, D) PMNs (black) and *T*. *vaginalis* (grey) were incubated for 2 hours in the presence of 20,000 U/ml of catalase (B) or 100 U/ml of DNase (D), and viability was determined as stated in Materials and methods. (C) Extracellular DNA was quantified with picogreen from supernatants after 2 hours incubation of PMNs with 100 nM PMA, with or without 100 U/ml of DNase. All data are represented as mean ± SD of triplicate wells and representative of 3 donors and 3 independent experiments. Underlying data can be found in S1 Data. MOI, multiplicity of infection; PMA, phorbol-myristate acetate; PMN, polymorphonuclear cell.
(TIF)

S5 Fig Quality controls for *T*. *vaginalis* cytotoxicity assays done with Cytochalasin D and wortmannin. (A) PMNs (black) and *T*. *vaginalis* (grey) were incubated for 2.3 hours in the presence of 2.5 ug/ml cytochalasin D or 50 ng/ml wortmannin, and viability was determined as described in Materials and methods. (B, C) Analysis of double positive events in cultures from *T*. *vaginalis* cytotoxicity assays in the presence of 2.5 ug/ml cytochalasin D (B), or 50 ng/ml wortmannin (C). Data shown are % CT^+^ among total CFSE^+^ cells. All data are represented as mean ± SD of triplicate wells and representative of 3 donors and 3 independent experiments. Underlying data can be found in S1 Data. CFSE, Carboxyfluorescein succinimidyl ester; CT, Cell Tracker; PMN, polymorphonuclear cell.
(TIF)

S6 Fig Heat-inactivated (dead) *T*. *vaginalis* are engulfed whole. *T*. *vaginalis* were labelled with CT and then incubated at 65 °C for 1 hour and confirmed dead. *T*. *vaginalis* were then cocultured with CFSE-labelled PMNs at identical conditions to those shown in Fig 3, and analyzed by imaging flow cytometry. To quantitatively compare the CFSE^+^CT^+^ double positive events in experiments using live versus heat-inactivated parasites, analysis of the intensity and measure of circular distribution of CT signal within CFSE^+^ cells was performed. The data show that CFSE^+^CT^+^ events from cocultures of PMNs with live parasites contain a lower intensity and more uneven (noncircular distribution) of CT^+^ signal, while those from cocultures of PMNs with dead parasites contain a higher intensity and a more circular distribution of CT^+^ signal, consistent with engulfment of whole parasites. (B) Dead (heat-inactivated) *T*. *vaginalis* were also cocultured with CFSE-labelled PMNs at identical conditions to those shown in Fig 1 and analyzed by flow cytometry. CT, Cell Tracker; CFSE, Carboxyfluorescein succinimidyl ester; PMN, polymorphonuclear cell.
(TIF)

S7 Fig *T*. *vaginalis* membrane material is not passively uptaken by nonphagocytic cells. Jurkats cells were incubated at MOI 0.1 with Alexa-488–labelled *T*. *vaginalis* as in Fig 4. Videos were monitored for transfer of green signal to Jurkat cells, which was never detected. Green signal never deviated from *T*. *vaginalis* cells. Images are representative of at least 3 parasites each from 3 independent experiments. MOI, multiplicity of infection.
(TIF)

S8 Fig Human serum factors, but not serine proteases, are required for phagocytosis of heat-inactivated (dead) *T*. *vaginalis*. (A) *T*. *vaginalis* were labelled with CT and then rendered dead using heat inactivation at 65 °C for 1 hour. Dead *T*. *vaginalis* were then cocultured with PMNs using conditions identical to Fig 5C. Cells were analyzed using imaging flow cytometry to determine the percent of *T*. *vaginalis* that were CT^+^CFSE^+^, and quantitatively determined as internal to PMNs as described in Materials and methods. Four donors and 4 independent experiments were analyzed for the percentage of *T*. *vaginalis* that were phagocytosed compared to vehicle control. (B) Representative images of phagocytosed *T*. *vaginalis* in the presence of 1-mM AEBSF are shown. (C) Dead *T*. *vaginalis* were cocultured with PMNs using conditions identical to Fig 6C (MOI 0.125). Then, percentage of phagocytosed *T*. *vaginalis* was determined by calculating the percent of CT^+^ cells that were CT^+^CFSE^+^. Underlying data can be found in S1 Data. CT, Cell Tracker; CFSE, Carboxyfluorescein succinimidyl ester; MOI, multiplicity of infection; PMN, polymorphonuclear cell.
(TIF)

S9 Fig Quality controls for *T*. *vaginalis* cytotoxicity assays done with Fc-blocking reagent. (A) PMNs (black) and *T*. *vaginalis* (grey) were incubated for 2.3 hours in the presence of 8-ug/ml Fc-blocking reagent or BSA, and viability was determined as described in Materials and methods. All data are represented as mean ± SD of triplicate wells. Underlying data can be found in S1 Data. BSA, bovine serum albumin; Fc, fragment crystallizable; PMN, polymorphonuclear cell.
(TIF)

S1 Data Raw numerical data. All individual data underlying summary data shown in bar graph format throughout the manuscript are shown and organized by tabs for each figure or supporting figure.
(XLSX)

S1 FCSfile FCS file for Fig 1B_Tv. FCS, fluorescence correlation spectroscopy.
(FCS)

S2 FCSfile FCS file for Fig 1B_PMN. FCS, fluorescence correlation spectroscopy.
(FCS)

S3 FCSfile FCS file for Fig 1B_Co-Culture. FCS, fluorescence correlation spectroscopy.
(FCS)

S4 FCSfile FCS file for S6B Fig. FCS, fluorescence correlation spectroscopy.
(FCS)

S5 FCSfile FCS file for Fig 6A_2ndary. FCS, fluorescence correlation spectroscopy.
(FCS)

S6 FCSfile FCS file for Fig 6A_HSerum. FCS, fluorescence correlation spectroscopy.
(FCS)

S7 FCSfile FCS file for Fig 6A_unstained. FCS, fluorescence correlation spectroscopy.
(FCS)

S8 FCSfile FCS file for Fig 6B_unstained. FCS, fluorescence correlation spectroscopy.
(FCS)

S9 FCSfile FCS file for Fig 1B_isotype. FCS, fluorescence correlation spectroscopy.
(FCS)

S10 FCSfile FCS file for Fig 1B_iC3. FCS, fluorescence correlation spectroscopy.
(FCS)

S1 Video PMNs swarm and trogocytose *T*. *vaginalis* before parasite death. PMN, polymorphonuclear cell.
(MP4)

S2 Video PMNs swarm and trogocytose *T*. *vaginalis* before parasite death. PMN, polymorphonuclear cell.
(MP4)

S3 Video PMNs internalize *T*. *vaginalis* fragments before parasite death. PMN, polymorphonuclear cell.
(MP4)

## Abbreviations

ADCC: antibody-dependent cell-mediated cytotoxicity
BSA: bovine serum albumin
CDD: contact-dependent degranulation
CFSE: Carboxyfluorescein succinimidyl ester
CT: Cell Tracker
Fc: fragment crystallizable
FCS: fluorescence correlation spectroscopy
HBSS: Hank’s Balanced Salt Solution
HPV: human papilloma virus
IL-8: interleukin 8
LDH: lactate dehydrogenase
mAb: monoclonal antibody
MOI: multiplicity of infection
NET: neutrophil extracellular trap
PFA: paraformaldehyde
PI: Propidium Iodide
PI3K: phosphoinositide 3-kinase
PMA: phorbol-myristate acetate
PMN: polymorphonuclear cell
ROS: reactive oxygen species
SD: standard deviation
STI: sexually transmitted infection
