# Supplementary figures and images for: Neutrophils kill the parasite Trichomonas vaginalis using trogocytosis
Source: PLoS Biol. 2018 Feb 6;16(2):e2003885. doi: 10.1371/journal.pbio.2003885 (PMC5815619; doi:10.1371/journal.pbio.2003885)

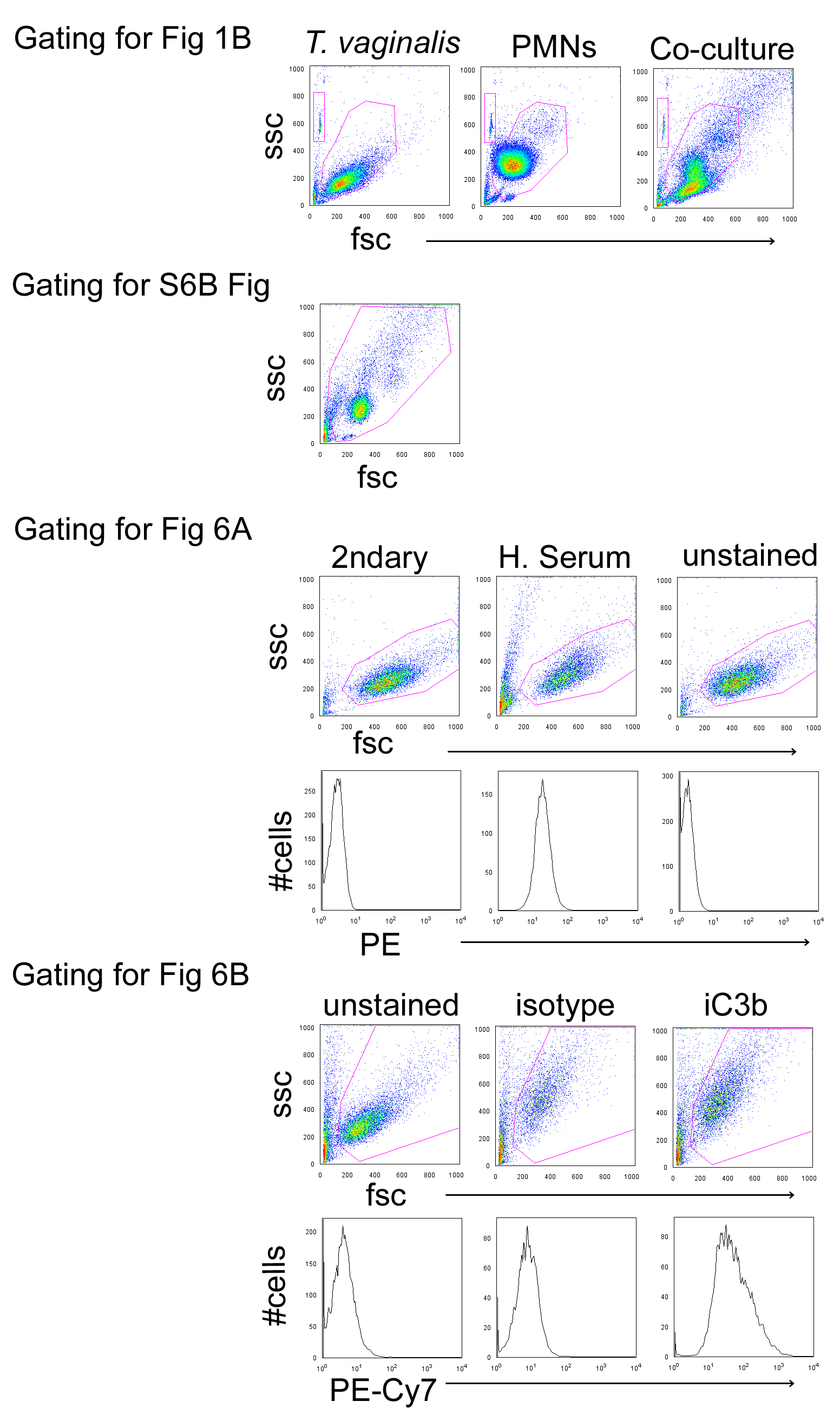

Supplement: S1 Fig — Gating strategies defining populations shown in FACS plots in Figs 1B, 6A and 6B and S6B using FCS files available as S1–S10 FCSfiles are shown. FACS, fluorescence-activated cell sorting; FCS, fluorescence correlation spectroscopy. (TIF) [file pbio.2003885.s001.tif]

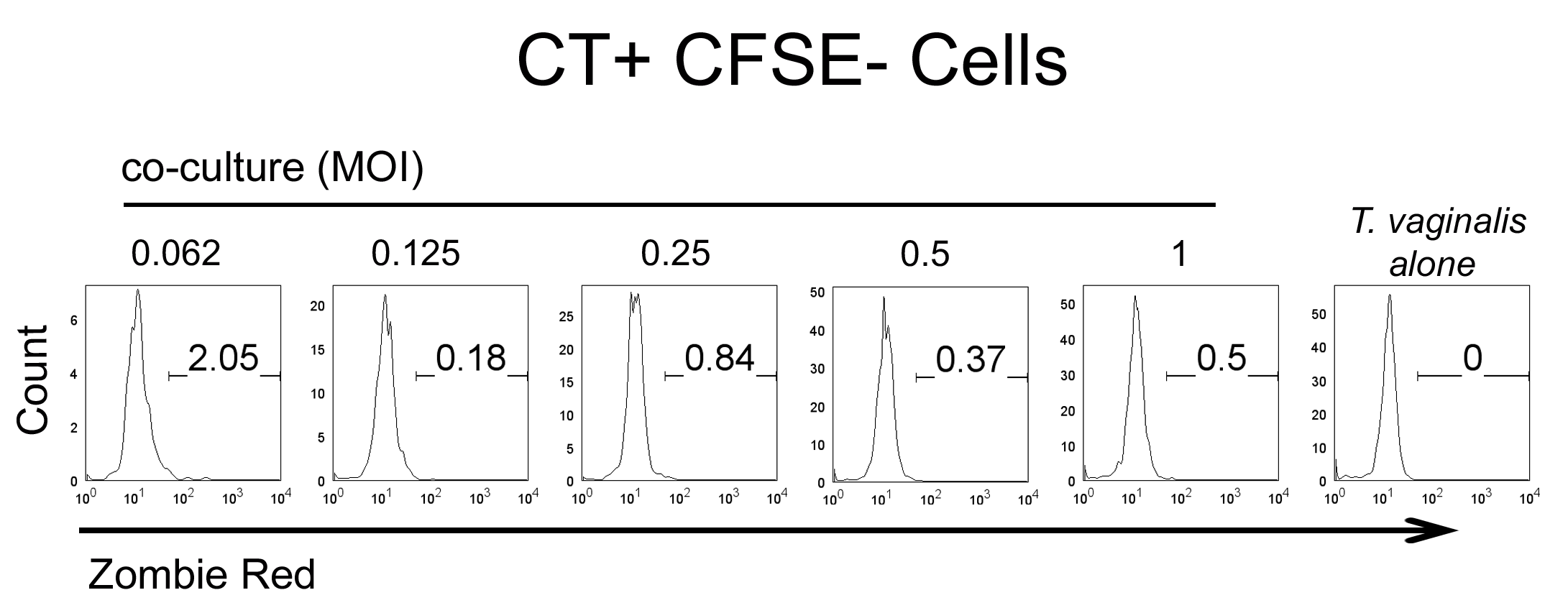

Supplement: S2 Fig — (A) To ensure that our live cell gate was accurate for flow cytometry–based cytolysis experiments, we labelled cells and cocultured as described in Fig 1. Then, all wells were stained with Zombie Red dead cell exclusion dye. The Zombie Red+ gate was set based on live T. vaginalis alone. We observed ≥98% of cells to be Zombie Red negative at all MOIs tested, indicating that surviving parasites in our assays are viable. CFSE, Carboxyfluorescein succinimidyl ester; CT, Cell Tracker; MOI, multiplicity of infection; PMN, polymorphonuclear cell. (TIF) [file pbio.2003885.s002.tif]

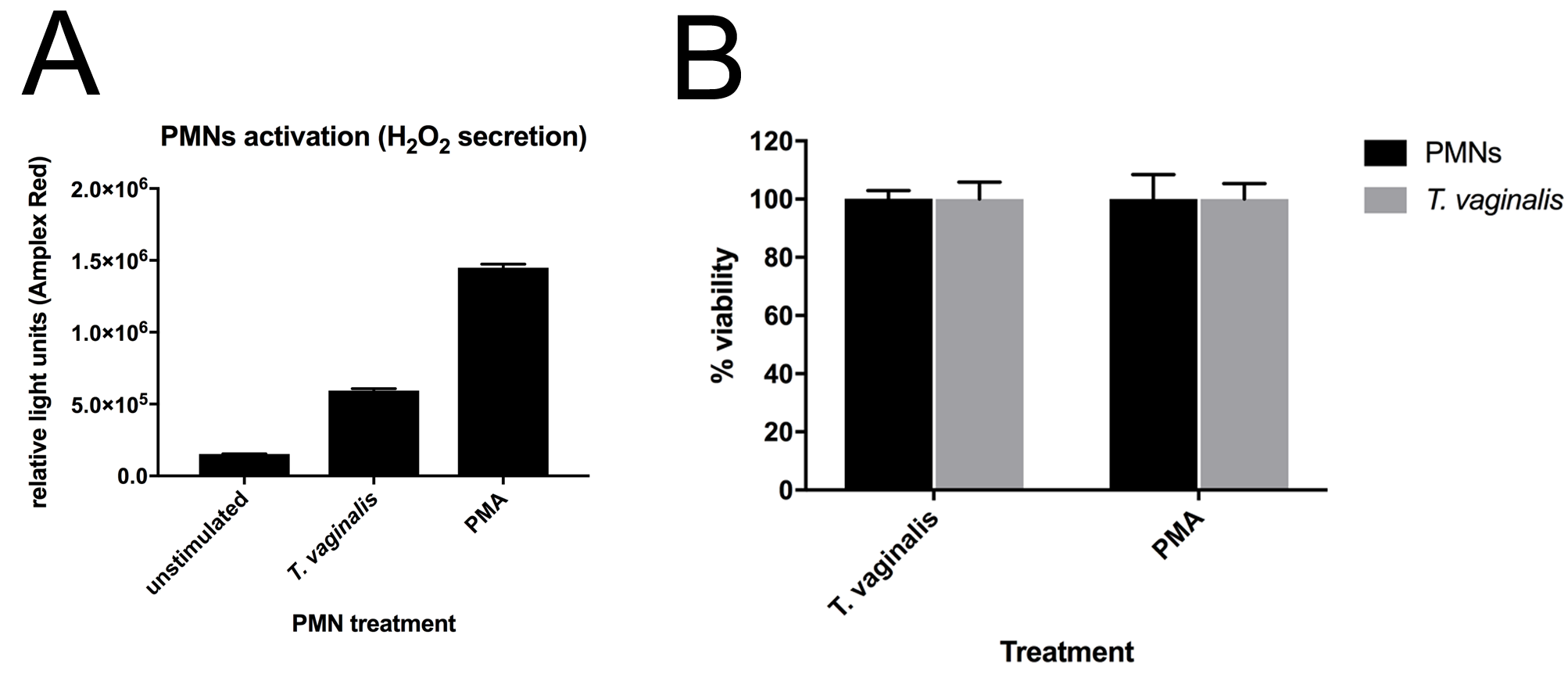

Supplement: S3 Fig — (A) PMN degranulation, as assessed by Amplex Red indicator of H2O2 activity, in the presence of T. vaginalis MOI 0.125 or 100 nM PMA, is shown. (B) PMNs (black) and T. vaginalis (grey) were incubated for 2 hours in the presence of T. vaginalis MOI 0.125 or 100 nM PMA, and viability was determined as described in Materials and methods. All data are represented as mean ± SD of triplicate wells and representative of 3 donors and 3 independent experiments. Underlying data can be found in S1 Data. MOI, multiplicity of infection; PMA, phorbol-myristate acetate; PMN, polymorphonuclear cell. (TIF) [file pbio.2003885.s003.tif]

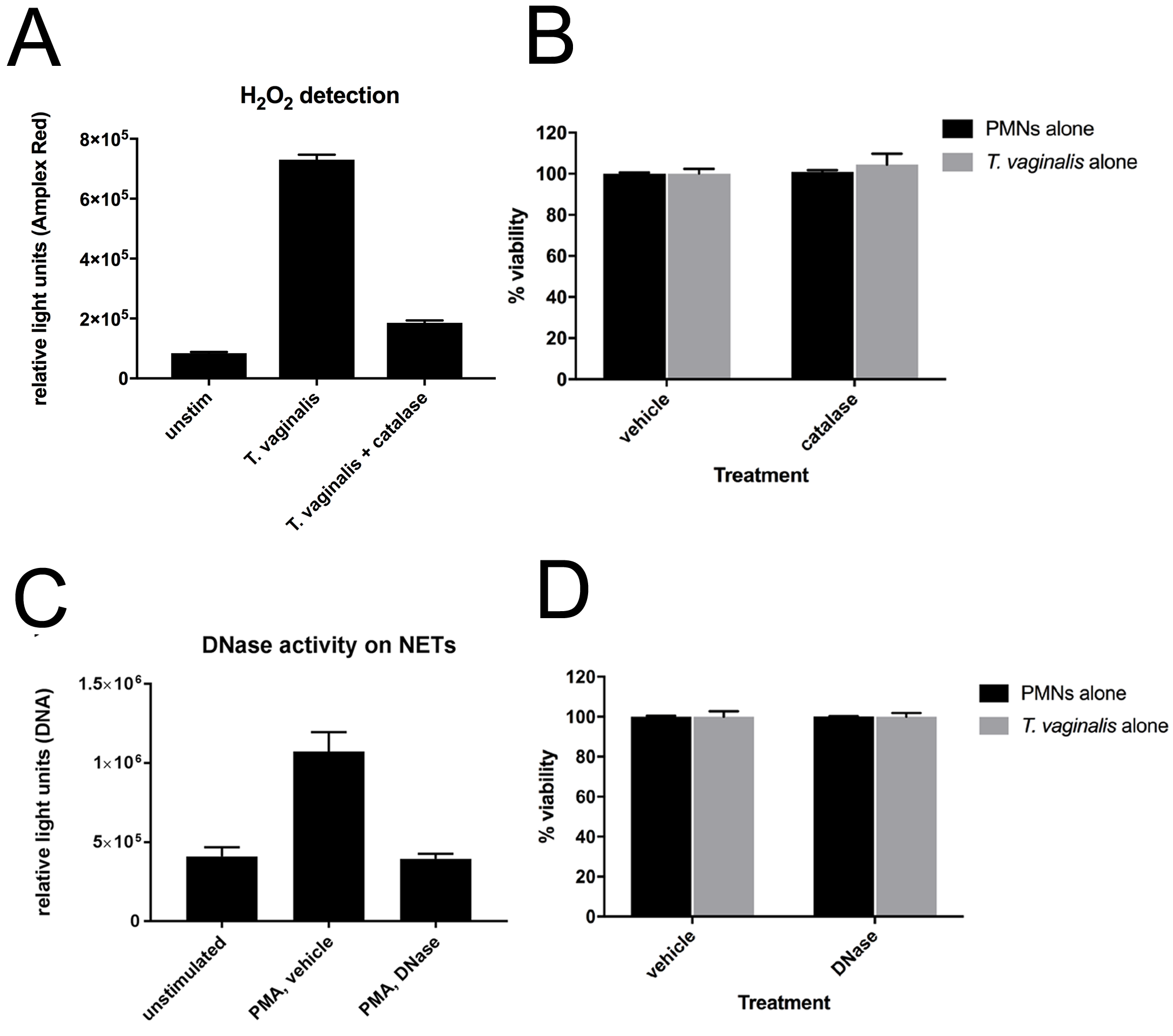

Supplement: S4 Fig — (A) H2O2 secretion, as assessed by Amplex Red indicator, was measured in wells of PMNs treated with MOI 0.125 T. vaginalis with or without 20,000 U/ml Catalase. (B, D) PMNs (black) and T. vaginalis (grey) were incubated for 2 hours in the presence of 20,000 U/ml of catalase (B) or 100 U/ml of DNase (D), and viability was determined as stated in Materials and methods. (C) Extracellular DNA was quantified with picogreen from supernatants after 2 hours incubation of PMNs with 100 nM PMA, with or without 100 U/ml of DNase. All data are represented as mean ± SD of triplicate wells and representative of 3 donors and 3 independent experiments. Underlying data can be found in S1 Data. MOI, multiplicity of infection; PMA, phorbol-myristate acetate; PMN, polymorphonuclear cell. (TIF) [file pbio.2003885.s004.tif]

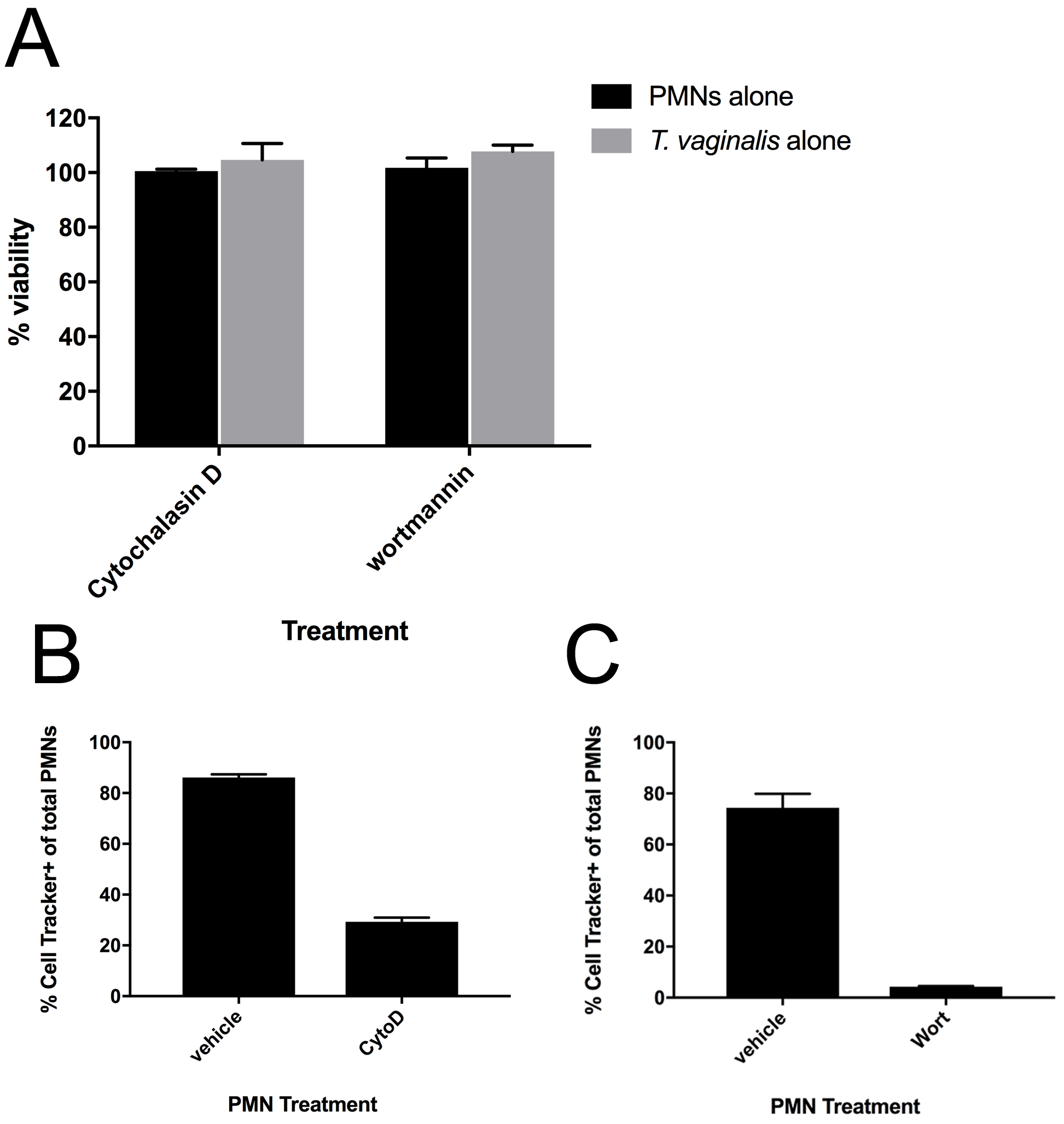

Supplement: S5 Fig — (A) PMNs (black) and T. vaginalis (grey) were incubated for 2.3 hours in the presence of 2.5 ug/ml cytochalasin D or 50 ng/ml wortmannin, and viability was determined as described in Materials and methods. (B, C) Analysis of double positive events in cultures from T. vaginalis cytotoxicity assays in the presence of 2.5 ug/ml cytochalasin D (B), or 50 ng/ml wortmannin (C). Data shown are % CT+ among total CFSE+ cells. All data are represented as mean ± SD of triplicate wells and representative of 3 donors and 3 independent experiments. Underlying data can be found in S1 Data. CFSE, Carboxyfluorescein succinimidyl ester; CT, Cell Tracker; PMN, polymorphonuclear cell. (TIF) [file pbio.2003885.s005.tif]

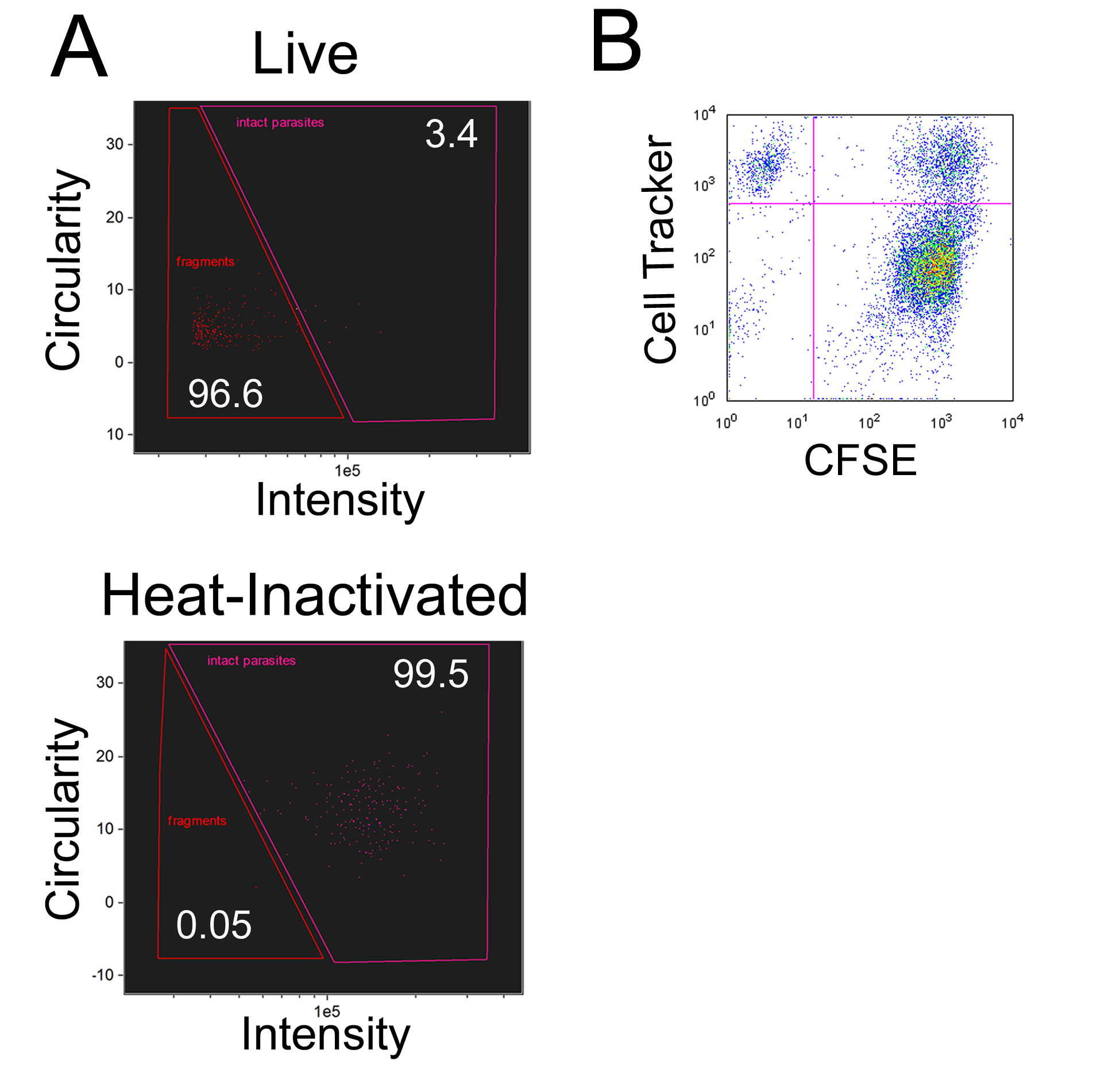

Supplement: S6 Fig — T. vaginalis were labelled with CT and then incubated at 65 °C for 1 hour and confirmed dead. T. vaginalis were then cocultured with CFSE-labelled PMNs at identical conditions to those shown in Fig 3, and analyzed by imaging flow cytometry. To quantitatively compare the CFSE+CT+ double positive events in experiments using live versus heat-inactivated parasites, analysis of the intensity and measure of circular distribution of CT signal within CFSE+ cells was performed. The data show that CFSE+CT+ events from cocultures of PMNs with live parasites contain a lower intensity and more uneven (noncircular distribution) of CT+ signal, while those from cocultures of PMNs with dead parasites contain a higher intensity and a more circular distribution of CT+ signal, consistent with engulfment of whole parasites. (B) Dead (heat-inactivated) T. vaginalis were also cocultured with CFSE-labelled PMNs at identical conditions to those shown in Fig 1 and analyzed by flow cytometry. CT, Cell Tracker; CFSE, Carboxyfluorescein succinimidyl ester; PMN, polymorphonuclear cell. (TIF) [file pbio.2003885.s006.tif]

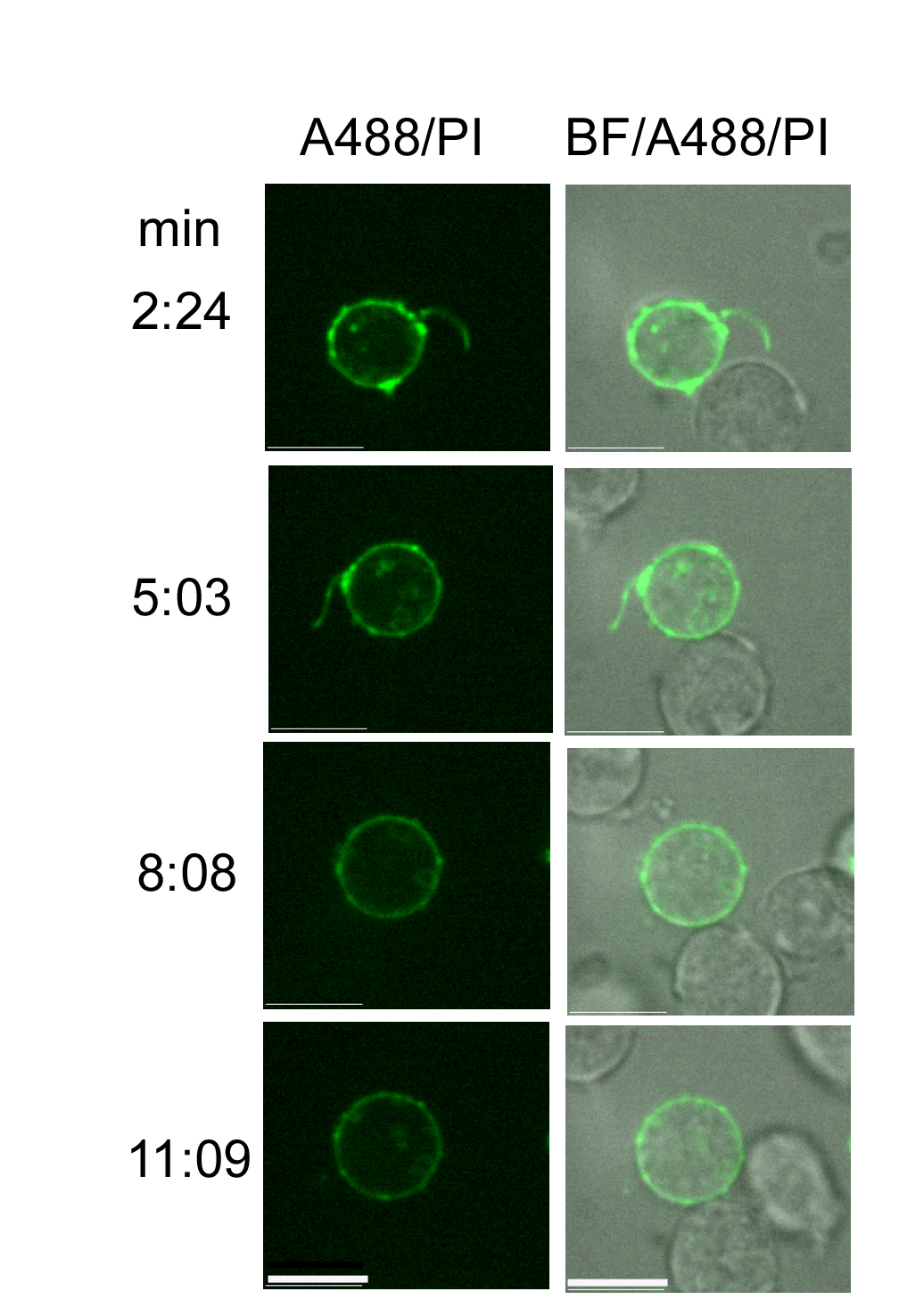

Supplement: S7 Fig — Jurkats cells were incubated at MOI 0.1 with Alexa-488–labelled T. vaginalis as in Fig 4. Videos were monitored for transfer of green signal to Jurkat cells, which was never detected. Green signal never deviated from T. vaginalis cells. Images are representative of at least 3 parasites each from 3 independent experiments. MOI, multiplicity of infection. (TIF) [file pbio.2003885.s007.tif]

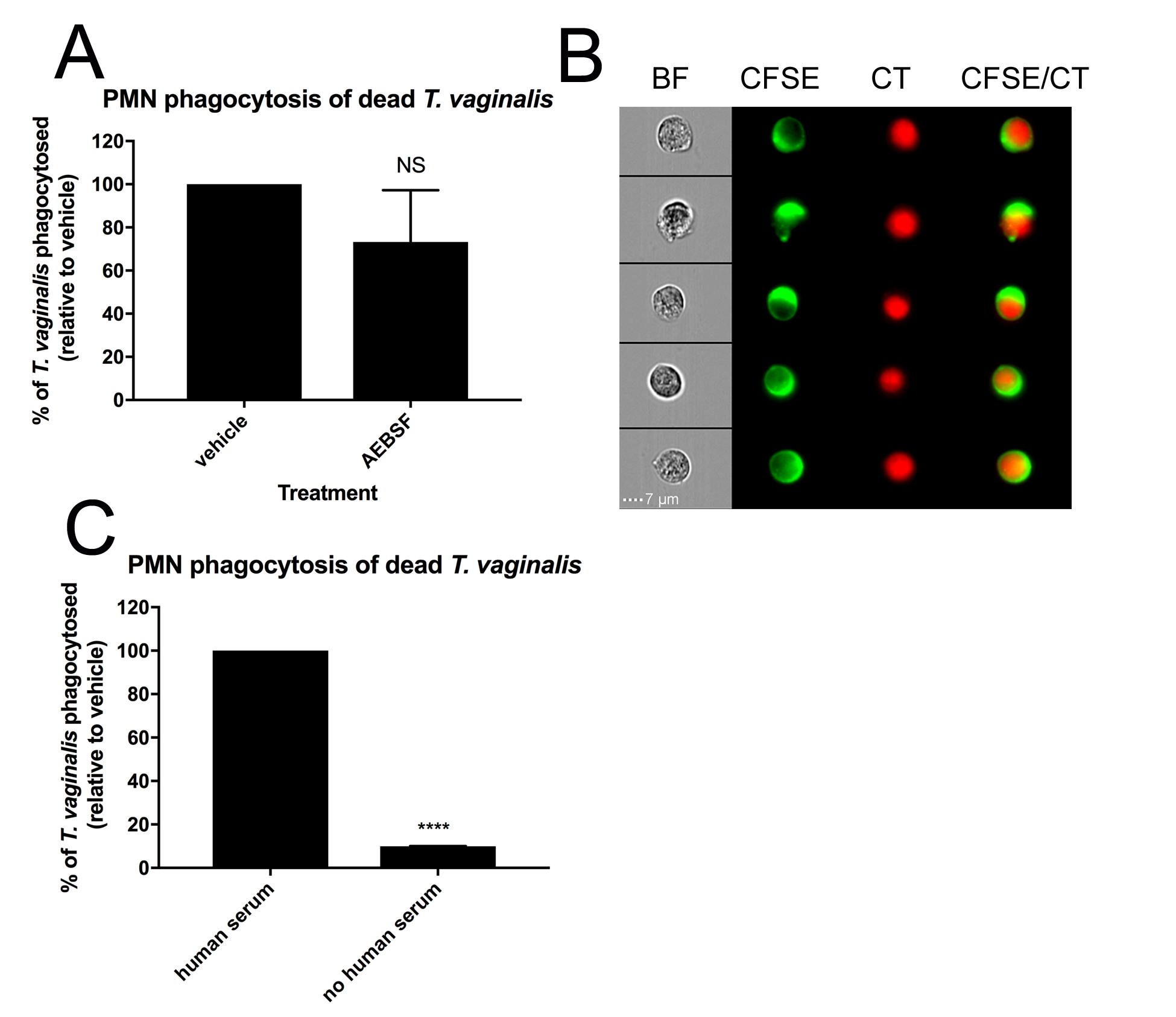

Supplement: S8 Fig — (A) T. vaginalis were labelled with CT and then rendered dead using heat inactivation at 65 °C for 1 hour. Dead T. vaginalis were then cocultured with PMNs using conditions identical to Fig 5C. Cells were analyzed using imaging flow cytometry to determine the percent of T. vaginalis that were CT+CFSE+, and quantitatively determined as internal to PMNs as described in Materials and methods. Four donors and 4 independent experiments were analyzed for the percentage of T. vaginalis that were phagocytosed compared to vehicle control. (B) Representative images of phagocytosed T. vaginalis in the presence of 1-mM AEBSF are shown. (C) Dead T. vaginalis were cocultured with PMNs using conditions identical to Fig 6C (MOI 0.125). Then, percentage of phagocytosed T. vaginalis was determined by calculating the percent of CT+ cells that were CT+CFSE+. Underlying data can be found in S1 Data. CT, Cell Tracker; CFSE, Carboxyfluorescein succinimidyl ester; MOI, multiplicity of infection; PMN, polymorphonuclear cell. (TIF) [file pbio.2003885.s008.tif]

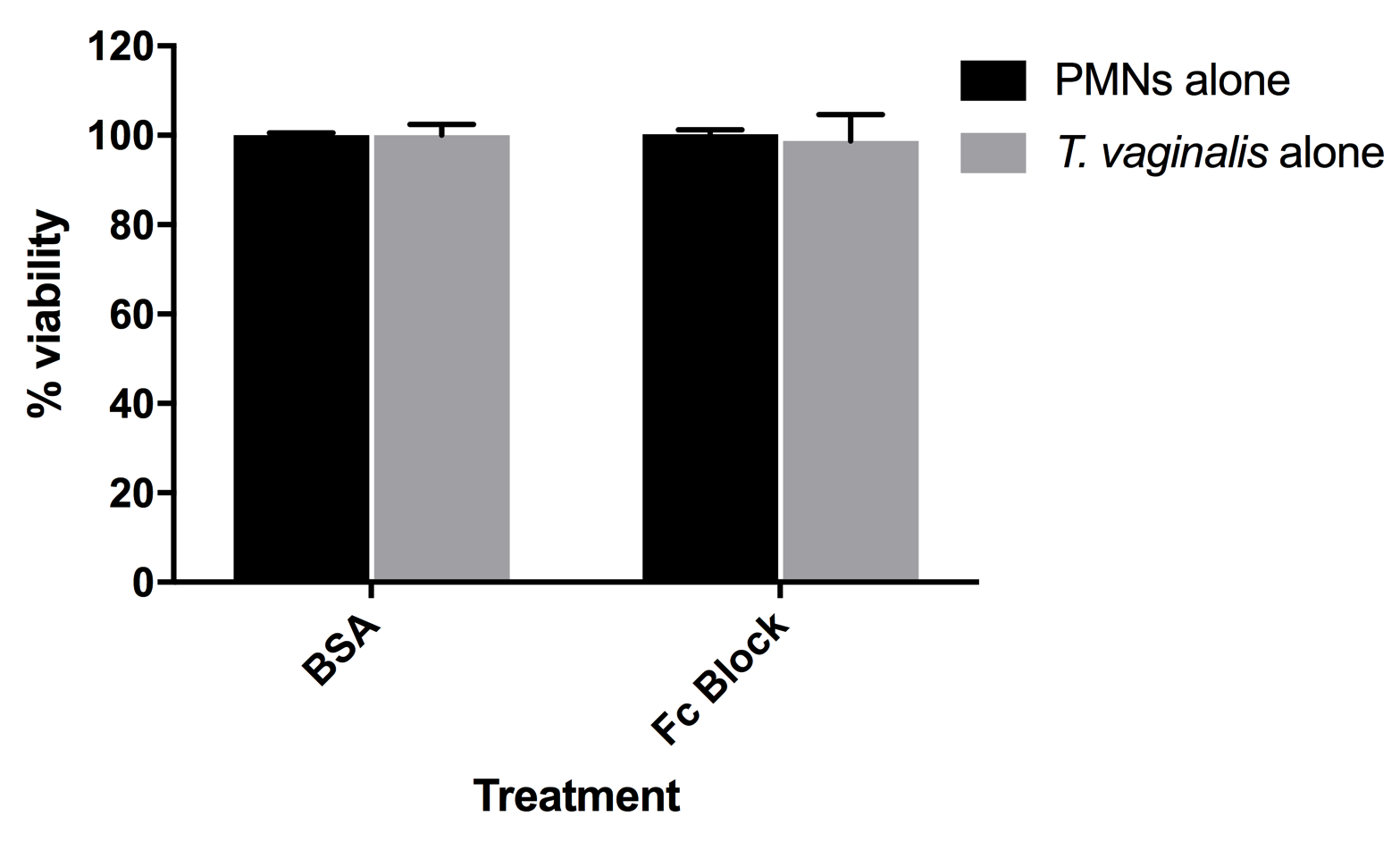

Supplement: S9 Fig — (A) PMNs (black) and T. vaginalis (grey) were incubated for 2.3 hours in the presence of 8-ug/ml Fc-blocking reagent or BSA, and viability was determined as described in Materials and methods. All data are represented as mean ± SD of triplicate wells. Underlying data can be found in S1 Data. BSA, bovine serum albumin; Fc, fragment crystallizable; PMN, polymorphonuclear cell. (TIF) [file pbio.2003885.s009.tif]
